# Pathway Evolution Through a Bottlenecking‐Debottlenecking Strategy and Machine Learning‐Aided Flux Balancing

**DOI:** 10.1002/advs.202306935

**Published:** 2024-02-06

**Authors:** Huaxiang Deng, Han Yu, Yanwu Deng, Yulan Qiu, Feifei Li, Xinran Wang, Jiahui He, Weiyue Liang, Yunquan Lan, Longjiang Qiao, Zhiyu Zhang, Yunfeng Zhang, Jay D. Keasling, Xiaozhou Luo

**Affiliations:** ^1^ Shenzhen Key Laboratory for the Intelligent Microbial Manufacturing of Medicines, Shenzhen Institute of Advanced Technology Chinese Academy of Sciences Shenzhen 518055 P. R. China; ^2^ CAS Key Laboratory of Quantitative Engineering Biology, Shenzhen Institute of Synthetic Biology, Shenzhen Institute of Advanced Technology Chinese Academy of Sciences Shenzhen 518055 P. R. China; ^3^ Center for Synthetic Biochemistry, Shenzhen Institute of Synthetic Biology, Shenzhen Institutes of Advanced Technology Chinese Academy of Sciences Shenzhen 518055 P. R. China; ^4^ The Key Laboratory of Industrial Biotechnology, Ministry of Education, School of Biotechnology Jiangnan University Wuxi 214122 P. R. China; ^5^ University of Chinese Academy of Sciences Beijing 100049 P. R. China; ^6^ Shenzhen Infrastructure for Synthetic Biology, Shenzhen Institute of Synthetic Biology, Shenzhen Institute of Advanced Technology Chinese Academy of Sciences Shenzhen 518055 P. R. China; ^7^ Joint BioEnergy Institute Emeryville CA 94608 USA; ^8^ Biological Systems and Engineering Division Lawrence Berkeley National Laboratory Berkeley CA 94720 USA; ^9^ Department of Chemical and Biomolecular Engineering & Department of Bioengineering University of California Berkeley CA 94720 USA; ^10^ Novo Nordisk Foundation Center for Biosustainability Technical University of Denmark Kgs. Lyngby 2800 Denmark

**Keywords:** biofoundry, directed evolution, machine learning, pathway debottlenecking

## Abstract

The evolution of pathway enzymes enhances the biosynthesis of high‐value chemicals, crucial for pharmaceutical, and agrochemical applications. However, unpredictable evolutionary landscapes of pathway genes often hinder successful evolution. Here, the presence of complex epistasis is identifued within the representative naringenin biosynthetic pathway enzymes, hampering straightforward directed evolution. Subsequently, a biofoundry‐assisted strategy is developed for pathway bottlenecking and debottlenecking, enabling the parallel evolution of all pathway enzymes along a predictable evolutionary trajectory in six weeks. This study then utilizes a machine learning model, ProEnsemble, to further balance the pathway by optimizing the transcription of individual genes. The broad applicability of this strategy is demonstrated by constructing an *Escherichia coli* chassis with evolved and balanced pathway genes, resulting in 3.65 g L^−1^ naringenin. The optimized naringenin chassis also demonstrates enhanced production of other flavonoids. This approach can be readily adapted for any given number of enzymes in the specific metabolic pathway, paving the way for automated chassis construction in contemporary biofoundries.

## Graphical abstract

A biofoundry‐assisted strategy for pathway bottlenecking and debottlenecking enables the parallel evolution of all pathway enzymes along a predictable evolutionary trajectory. A machine learning model can further relax the epistasis of the evolved pathway by optimizing the corresponding promoter combinations. Above strategy contributes to an Escherichia coli chassis with evolved and balanced pathway genes.

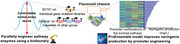

## 1 Introduction

Heterologous pathway engineering has emerged as a cornerstone in biosynthesis, playing a pivotal role in the production of a wide array of industrially relevant compounds, from pharmaceuticals to biofuels, thereby revolutionizing the landscape of synthetic biology and industrial biotechnology.^[^ ^1^ ^]^ The current strategies for improving these pathways primarily involve the optimization of enzyme expression levels, the enhancement of precursor supply, and the reduction of pathway bottlenecks.^[^ ^2^ ^]^ Advanced techniques such as dynamic control of pathway genes, computational design for enzyme discovery, and machine learning‐guided pathway optimization have been employed, offering unprecedented precision and efficiency in pathway improvement.^[^ ^1^, ^3^ ^]^ However, the process of evolving multiple pathway enzymes presents significant challenges, as it requires a delicate balance of multiple factors, including enzyme activity, stability, and specificity, and often involves navigating the complex and unpredictable landscape of protein engineering and metabolic flux optimization.^[^ ^4^ ^]^

Natural evolution typically proceeds through incremental improvements in the activity, stability, and specificity of a particular enzyme, one at a time.^[^ ^4^, ^5^ ^]^ This gradual process has often been attributed to high levels of epistasis, a phenomenon where the effects of genetic mutations are contingent upon other mutations or the broader genetic context, leading to a complex relationship between different mutations. A mutation that is advantageous in certain genetic settings might become harmful or even fatal in others. Thus, traits with high epistasis limit evolutionary potential, potentially reducing adaptability.^[^ ^4^, ^5^, ^6^ ^]^ For instance, most mutation combinations in multiple pathway enzymes can be detrimental or even lethal to specific species.^[^ ^7^ ^]^ The task of scanning all these combinations to identify the most beneficial mutations also exceeds the capacity of the population.^[^ ^5^, ^8^ ^]^ Metabolic control theory further suggests that minor improvements in one enzyme often render another enzyme the bottleneck of the pathway.^[^ ^6^, ^7^ ^]^ As a result, evolution has been regulated by nature to proceed at a slow pace, requiring millennia to augment an existing function or develop a new one.^[^ ^5b^ ^]^ This gradual adaptation process, which spans countless generations, is an impractical timeframe for industrial applications.^[^ ^5^, ^9^ ^]^

In recent decades, several strategies have been proposed to accelerate the evolution of multienzymes in a specific pathway, including MAGE^[^ ^9a^ ^]^ and BacORep.^[^ ^10^ ^]^ These methods leverage in vivo mutagenesis to streamline library construction and implement continuous multi‐generation culturing to emulate nature's gradual adaptation process.^[^ ^6^, ^11^ ^]^ Despite these advancements, the achieved improvements have been relatively incremental, and the final product titers remain low.^[^ ^10^, ^12^ ^]^ This phenomenon highlights a key challenge in the laboratory‐scale directed evolution for industrial applications: the feasible timeframe and available generations are considerably less than what nature typically requires for significant evolutionary progress. To overcome these limitations and further accelerate the directed evolution process in a rugged landscape, a more effective approach is needed. For instance, one can change the landscape to make it smoother, or one can find a known evolutionary trajectory. That means the ideal approach would 1) operate within a known evolutionary space for each enzyme to enable substantial improvements within a practical timeframe; 2) employ a single sensor and assay for all pathway enzymes in the known evolutionary space; 3) support parallel and iterative operations with minimal human intervention.

**Figure 1:**
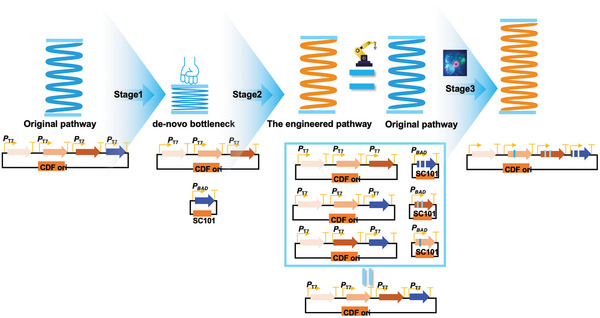
Pathway bottlenecking‐and‐debottlenecking strategy in this study. This pathway engineering strategy comprises three stages. Design the artificial naringenin pathway bottleneck by expressing the individual naringenin gene into the low‐copy plasmid with the weak promoter (stage 1). Eliminate the de‐novo bottleneck of the naringenin pathway by screening the candidate mutants, which produce a similar naringenin production to that of the original pathway (stage 2). Put mutants of the individual genes back into the original pathway and further balance the metabolic flux by artificial‐intelligence‐mediated promoter engineering (stage 3).

Here, we initially illustrated the complex and rugged evolutionary landscape of multiple genes within a heterologous pathway. Subsequently, we introduced a method based on a bottlenecking and debottlenecking strategy (**Figure** **1**). This approach not only reduced the ruggedness of the evolutionary landscape for these enzymes, but also provided a predictable evolutionary trajectory for them. The method employed the concentration of the final product as the sole selection criteria for the evolution of all pathway enzymes, thereby obviating the need for multiple distinct assays for each enzyme and broadening the method's universal applicability. Moreover, this approach permits the parallel evolution of individual genes, facilitated by an automation‐based method to mitigate labor‐intensive work, and is further enhanced by an AI algorithm for additional pathway optimization. As a result, naringenin biosynthesis has been amplified to over 3 g L^−1^, underscoring the potential of this method for future multienzyme engineering endeavors.

## 2 Results and Discussion

### 2.1 Investigation of Epistasis Among Heterologous Pathway Genes in Naringenin Biosynthesis

**Figure 2:**
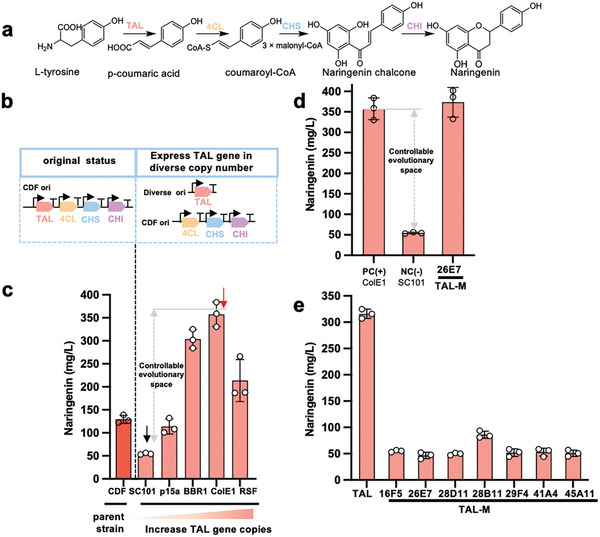
Directed evolution of TAL as an example to evaluate potential epistasis of the heterologous naringenin biosynthetic pathway a) Naringenin biosynthesis pathway: Tyrosine is transformed to naringenin by four enzymes: tyrosine ammonia‐lyase (TAL), 4‐coumarate‐CoA ligase (4CL), chalcone synthase (CHS) and chalcone isomerase (CHI). b) Various constructs were tested for narinengin production, with its original status as all four genes were under the control of PT7 in the same pCDF plasmid and the dual‐plasmid system where the individual gene under the control of PBAD promoter was placed on plasmids with diverse copy numbers and co‐expressed with the other three genes on pCDF. c) Influence of different plasmid copy numbers on naringenin production. The TAL gene was placed in pBbS8C (SC101 replicon, 5–10 copies), pBbA8c (p15a replicon, 10–15 copies), pBbB8a (BBR1 replicon, 17–20 copies), pBbE5K (ColE1 replicon, 20–30 copies) and pRSF (RSF replicon, 100 copies). The parent strain contains the pCDF plasmid (CDF replicon, 20 copies) with TAL, 4CL, CHS, and CHI genes. The highest producers are labeled with red arrows and the artificial pathway bottlenecks are marked with blank arrows. The gray arrows indicate the expected evolutionary space. Naringenin concentrations were determined by HPLC in biological triplicates and the error bars represent the standard deviation. d) TAL, when present in low copy number plasmid, was successfully evolved to produce the same amount of naringenin as the positive control. TAL‐26E7 is the candidate TAL mutant with the highest naringenin production, PC (+) strain, positive control strain containing pBbE5K‐TAL, and pCDF‐4CL‐CHS‐CHI. NC (‐), negative control strain containing plasmid pBbS8C‐TAL and pCDF‐4CL‐CHS‐CHI. e) Investigating the potential epistasis of the TAL gene. The TAL gene and its mutants in pBbE5K (ColE1 replicon, 20–30 copies) plasmid were transformed into competent cells harboring plasmid pCDF‐4CL‐CHS‐CHI (4SI) or pCDF‐4CL‐11C1‐CHS‐9H9‐CHI (4SI‐M), respectively. Naringenin concentrations were determined by HPLC in biological triplicates and the error bars represent the standard deviation.

To investigate the prevalence of epistasis among heterologous pathway genes in naringenin biosynthesis, we first assembled four well‐characterized pathway genes (**Figure** **2**a) encoding tyrosine ammonia‐lyase (TAL) from *Rhodotorula toruloides*, 4‐coumarate‐CoA ligase (4CL) from *Petroselium crispum*, chalcone synthase (CHS) from *Petunia x hybrida* and chalcone isomerase (CHI) from *Medicago sativa*, under the control of four individual T7 RNA polymerase promoters (P_T7_) and inserted them into a pCDF vector to form plasmid pCDF‐T4SI which has been demonstrated to produce a high‐level of naringenin (Figures 1 and 2b).^[^ ^13^ ^]^ This plasmid was then transformed into *Escherichia coli* BL21(DE3), and the production of naringenin was quantified by high‐performance liquid chromatography (HPLC) at 129.67 mg L^−1^ after a 48‐h expression in a 96‐well plate culture (Figure 2c).

Given that certain mutations may be deleterious in one context but advantageous in another, thereby allowing the fittest genotypes to be accessed only through the accumulation of mutations in a specific sequence,^[^ ^4a,c^ ^]^ we aimed to identify a beneficial mutant for TAL and subsequently assess its fitness under different conditions. To start, we established a starting point for directed evolution of TAL by examining a P_BAD_ promoter‐driven TAL on plasmids with different copy numbers, including pBbS8C (SC101 replicon, 5–10 copies), pBbA8c (p15a replicon, 10–15 copies), pBbB8a (BBR1 replicon, 17–20 copies), pBbE5K (ColE1 replicon, 20–30 copies) and pRSF (RSF replicon, 100 copies),^[^ ^14^ ^]^ whereas we kept the other three genes in the pCDF plasmid under the control of P_T7_ promoter (pCDF‐4SI) (Figure 1b,c). Then a random mutagenesis library of TAL, TAL_lib_, was integrated into pBbE5K, which showed the highest naringenin production of 357.66 mg L^−1^, to obtain a library pBbE5K‐TAL_lib_ for its directed evolution. A previously reported Al^3+^ assay was used to screen for variants with higher naringenin titers. However, after evaluating more than 3000 variants, no hit showed a higher production, suggesting the complicated epistasis might trapped the evolution to its local maxima.

To obtain better TAL mutants to evaluate the epistasis in the heterologous naringenin pathway, we then placed TAL_lib_ library on the plasmid with the SC101 origin, which is maintained at a low copy number in cells. The resulting plasmid library, pBbS8C‐TAL_lib_, allowed a more manageable evolutionary trajectory for the directed evolution of TAL, as improvement of TAL to reach the same naringenin production as in pBbE5K should not encounter problems such as intermediate toxicity or inter‐pathway regulation. When it was co‐transformed with pCDF‐4SI for Al^3+^ assay screening (Figure 2b; Figure S1, Supporting Information), 179 variants exhibited stronger Al^3+^ assay signals than their corresponding control, from which the top 7 were further validated by HPLC to confirm their enhanced naringenin production. Their mutation sites were revealed by sequencing (Table S3, Supporting Information). The kinetic parameters of the TAL mutant from the best naringenin producer, TAL‐26E7, were then assessed with an in vitro assay.^[^ ^15^ ^]^ The *k_cat_*/*K*_M_ for TAL‐26E7 was 1158 mm ^−1^⋅s^−1^ (**Table** **1**), which was 3.86‐fold higher than its wild‐type counterpart. These results suggest that TAL‐26E7, when present on a low‐copy plasmid, is indeed a beneficial mutant for both enzyme activity and naringenin production (Figure 2d).

**Table 1:** Kinetic properties of naringenin‐associate genes and their mutants.

Genes	*K_M_* (mM)	*k_cat_* (s^−1^)	*k_cat_*/*K_M_* (mM^−1^s^−1^)
TAL	0.38	114.00	300.00
TAL‐26E7(H174Q)	2.09	2416.00	1158.20
4CL	0.65	3.01*10^6^	4.63*10^3^
4CL‐11C1 (L66P)	0.06	5.75*10^6^	9.58*10^3^

We then placed the wild‐type TAL and all 7 selected TAL variants with the improved activities on pBbE5K to evaluate their performance and investigate the potential epistasis. The resulting plasmids were co‐transformed with pCDF‐4SI and their naringenin productions were monitored by HPLC. Although pBbE5K‐TAL showed a high naringenin titer of 357.66 mg L^−1^, the production of naringenin was lower for all TAL variants, with the highest being 86.22 mg L^−1^ (TAL‐28B11) and lowest being 46.58 mg L^−1^ (TAL‐26E7) (Figure 2e). This provides compelling evidence that when TAL is present in a medium to high copy plasmid such as pBbE5K, epistasis could potentially obscure the identification of a beneficial mutant with enhanced naringenin production. This suggests that epistasis could increase the likelihood of the pathway becoming trapped at local maxima in the fitness landscape due to the acquisition of mutations in an unfavorable sequence.^[^ ^6a^ ^]^ These findings also elucidate why pathway evolution often results in minimal or no improvement.^[^ ^4a,c^ ^]^

### 2.2 Parallel and Sequential Evolution of Pathway Enzymes Using a Biofoundry

**Figure 3:**
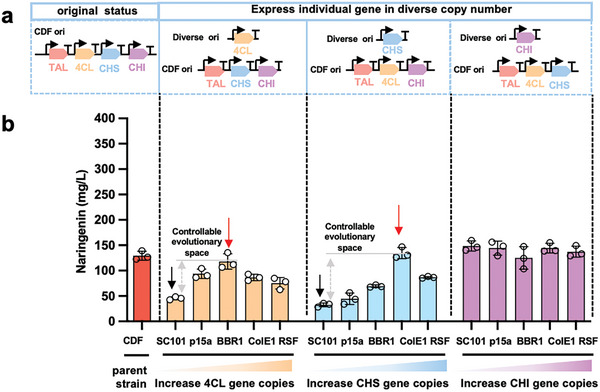
Increased naringenin production by introducing de‐novo pathway bottleneck. (a) The various constructs tested for narinengin production, with its original status as all four genes were under the control of PT7 in the same pCDF plasmid and the dual‐plasmid system where individual gene under the control of PBAD promoter was placed on plasmids with different copy numbers and co‐expressed with the other three genes on pCDF. (b) Influence of different plasmid copy numbers on naringenin production. Individual genes were placed in pBbS8C (SC101 replicon, 5–10 copies), pBbA8c (p15a replicon, 10–15 copies), pBbB8a (BBR1 replicon, 17–20 copies), pBbE5K (ColE1 replicon, 20–30 copies) and pRSF (RSF replicon, 100 copies). Parent strain contains the pCDF plasmid (CDF replicon, 20 copies) with TAL, 4CL, CHS and CHI genes. The highest producers are labeled with red arrows, and the artificial pathway bottlenecks are marked with blank arrows. The gray arrows indicate the expected evolutionary space. Naringenin concentrations were determined by HPLC in biological triplicates, and the error bars represent the standard deviation.

The evolutionary landscapes for heterologous pathways, as evidenced by TAL mutants, can be complex and their directed evolution may frequently encounter local maxima. This phenomenon has also been illustrated in the case of TEM1 β‐lactamase. A variant of TEM1 β‐lactamase, possessing five mutations, exhibited the ability to cleave cefotaxime.^[^ ^4b^ ^]^ However, out of the 120 conceivable pathways leading to this 5‐mutant variant, a mere 7% are evolutionarily accessible, as the majority traverse fitness valleys where the mutation combinations diminish activity. Contrastingly, environmental alterations, which inherently reshape the fitness landscape, have been shown to offer an escape from these local maxima.^[^ ^4a^ ^]^ Thus, it becomes crucial to reconfigure the fitness landscape and pinpoint a distinct evolutionary trajectory that enables feasible enhancement of the entire pathway. The method employed should satisfy the following criteria: 1) A single assay and output for all pathway enzymes, eliminating the need for additional assay development; 2) The capability to perform the assay in a high‐throughput manner, ideally exceeding 10^3^–10^4^; and 3) The potential for iterative application of the method. In response to these requirements, we devised a method that modifies the environment and evolution landscape by reducing the expression level of each enzyme individually (**Figures** 2c and **3**b). This alteration creates a fitness landscape with a single, clear uphill trajectory, allowing recovery to the original activity level with high expression before any potential epistatic interactions occur. The final product of the pathway serves as an indicator of pathway performance (Figure S1, Supporting Information), which not only provides a direct measurement but also facilitates high‐throughput analysis using a biosensor, chemical sensor, or high‐throughput mass spectrometry. Lastly, we have developed a biofoundry‐based automation method to expedite the iterative rounds of evolution (Figure S2, Supporting Information).

**Figure 4:**
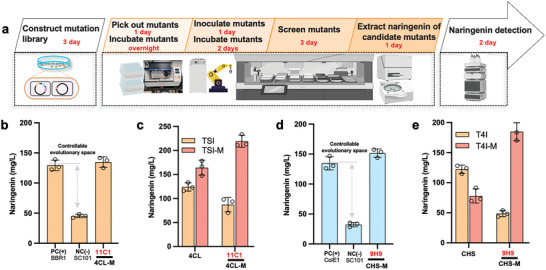
Biofoundray‐assisted engineering of 4CL and CHS genes in controllable evolutionary space, and investigation of inter‐gene epistasis. a) Automation workflow for high throughput screening of mutants of the de‐novo pathway bottleneck. This automation procedure contains four steps: 1) picking candidate mutants using a QPix 400 (Video S1, Supporting Information); 2) transferring the overnight culture into fresh fermentation broth (Video S2, Supporting Information); 3) screening candidate mutants using an Al3+ assay (Video S3, Supporting Information); and 4) analyzing naringenin titers using HPLC (Video S4, Supporting Information). b) 4CL was successfully evolved to produce the same amount of naringenin as the positive control. 11C1 is the candidate 4CL mutant with higher naringenin production, PC (+) strain, positive control strain containing pCDF‐TAL‐4CL‐CHS‐CHI. NC (‐), negative control strain containing pBbS8C‐4CL and pCDF‐TAL‐CHS‐CHI. c) Investigating the potential epistasis of 4CL gene. 4CL gene and the candidate mutant in pBbB8a plasmid were transformed into competent cells with plasmid pCDF‐TAL‐CHS‐CHI (TSI) and pCDF‐TAL‐26E7‐11C1‐CHS‐9H9‐CHI (TSI‐M), respectively. d) CHS was successfully evolved to produce the same amount of naringenin as the positive control. 9H9 is the candidate CHS mutant with higher naringenin production, PC (+) strain, positive control strain containing pBbE5K‐CHS and pCDF‐TAL‐4CL‐CHI. NC (‐), negative control strain containing pBbS8C‐CHS and pCDF‐4CL‐CHS‐CHI. (e) Investigate the potential epistasis of CHS gene. CHS gene and the candidate mutant in pBbE5K plasmid were transformed into competent cells with plasmid pCDF‐TAL‐CHS‐CHI (TSI) or pCDF‐TAL‐26E7‐11C1‐CHS‐9H9‐CHI (TSI‐M), respectively. Naringenin concentrations were determined by HPLC in biological triplicates and the error bars represent the standard deviation.

As an effective infrastructure to automate the “design‐build‐test‐learn” cycle, the biofoundry has been widely applied to increase experiment throughput and decrease human errors.^[^ ^16^ ^]^ Therefore, to enable the iterative evolution of naringenin pathway enzymes, we designed a biofoundry with a liquid handler system and a robotic arm‐accessible incubator, centrifuge, plate reader, and other accessories (complete list in Figure S2, Supporting Information), as well as a stand‐alone QPix 400 system for colony picking and a stand‐alone liquid chromatograph for product quantification. The automated workflow for the biofoundry was developed (Videos [Link], [Link], [Link], [Link], Supporting Information), starting with the inoculation of about 5000 clones for each enzyme from an agar plate containing a random mutagenesis library of the corresponding genes into a 96‐deep well plate with 0.8 mL MOPS medium (Figure S3 and Video S1, Supporting Information). The resulting 50 96‐well plates were then transferred into an automatic incubation system and incubated at 800 rpm and 30°C overnight (**Figure** **4**a). The cultures were then diluted (1% v/v) into 96‐deep well plates with 0.8 mL MOPS and incubated at 800 rpm and 30°C for 2 days (Figure S4 and Video S2, Supporting Information). The plates were then moved to an automatic centrifuge to separate the supernatant from the pellet, from which 100 µL supernatant was retrieved for the Al^3+^ assay (Figures S1 and S5 and Video S3, Supporting Information).^[^ ^17^ ^]^ An algorithm was developed to identify hits with higher Al^3+^ signals than that of the corresponding control. The pellets of these hits were resuspended, and an equal volume of ethanol was added to each well. The plates were then vortexed at 800 rpm for 1 min and then incubated for 30 min at room temperature to facilitate cell lysis and product extraction. The supernatant containing naringenin was collected by centrifugation for HPLC validation (Figure S6 and Video S4, Supporting Information). The mutants with higher titers by HPLC were then sequenced and used for downstream experiments. The whole automation process lasts for 2 weeks per round with a throughput of about 11 000 colonies (115 96‐deep wells) per run without a production schedule, allowing in theory the parallel evolution of 2 genes with 5000 colonies each or 1 gene with 10000 colonies each.

To evaluate the reliability of the automated workflow, we performed a head‐to‐head comparison between manual and automated experiments, monitoring three key indicators (OD_600_ for cell growth, Al^3+^ assay signal for primary screening accuracy, and naringenin concentration for extraction efficiency). Ten strains with different levels of naringenin titers from Figure 1 were selected for evaluation. It has been shown that no significant growth difference was observed between hand‐inoculated and incubated cultures versus the same strains processed using automation (Figure S7a, Supporting Information). The Al^3+^ assay (Figure S7b, Supporting Information) and the naringenin extraction assay (Figure S7c, Supporting Information) also demonstrated a high correlation between manual and automated experiments, with an R^2^ of 0.986 and 0.983, respectively (Figure S8, Supporting Information). Thus, the workflow was then used for the automated directed evolution of 4CL, CHS, and CHI with artificial bottlenecks. Before that, we assessed the evolvability and evolutionary ceiling for these three enzymes through processes that were similar to that of TAL (Figures 2c and 3b). The result indicated that 4CL and CHS were bottlenecks when their genes were expressed from a plasmid with SC101 origin (Figure 3b); they were evolvable as the reason for these bottlenecks was solely because of their insufficient expressions (Figure 3b). The activities of these enzymes whose genes were expressed from SC101 plasmids could be improved by at least 2.85‐ and 4.15‐fold, respectively (Figure 3b). Therefore, 4CL and CHS were sequentially evolved using the automated workflow (Figure 4a). Random mutagenesis libraries of each gene were constructed in the low copy pBbS8C (Figure 3). We expected that the increase in the enzyme activity should increase the naringenin production to the same level as if these enzymes were overexpressed since all other conditions remained the same. Therefore, the corresponding plasmid harboring the same gene with the origin that resulted in the highest titer was used as a positive control, as these titers should be the minimum reachable evolutionary outcome (Figures 3b and 4b,d). In total, 12 and 57 positive hits with higher Al^3+^ assay signals than the corresponding controls were identified for 4CL and CHS, respectively, from which the top 5 and 2 mutants, respectively, were further validated by HPLC to produce more naringenin. Their mutation sites were revealed by sequencing (Table S3, Supporting Information). The highest titers were obtained with mutants 4CL‐11C1 and CHS‐9H9 which were close to their corresponding positive control (Figure 4b,d), demonstrating the success of the artificial bottleneck strategy and implying the possibility of an evolutionary boundary by epistasis. Above results also demonstrated that the automation platform's high throughput and low error rate make it easily expandable for multiple rounds or iterative enzyme engineering, as well as the evolution of more complex pathways with multiple enzymes, especially when used in conjunction with a target molecule that has fluorescence or colorimetric reporting capabilities.^[^ ^16a^ ^]^ The naringenin titers of these mutants were 3.00‐, and 4.83‐fold enhanced in comparison with wild‐type 4CL and CHS, respectively (Figure 4b,d). We then evaluated the kinetic parameters of these mutants, and the results are summarized in Table 1. While *k_cat_s* were improved for all mutants, the *K*_M_ for CHS‐9H9 was slightly increased, indicating a weaker binding affinity for their substrates. The *k_cat_*/*K*_M_ for 4CL‐11C1 and CHS‐9H9 were 9583 and 1435 mM^−1^⋅s^−1^, which were 2.07‐ and 4.16‐fold increase compared with their wild‐type counterparts, respectively (Supplementary Tables S4–S6, Supporting Information). We also noticed that the mutated residues of TAL and CHS were located away from their catalytic centers, suggesting that the high throughput automation workflow could be used as an efficient tool to explore previously unknown sites (e.g., distal sites from the active center) that might not be predicted by rational design.

### 2.3 Investigation of Inter‐Gene Epistasis

While high levels of epistasis are typically viewed as constraints on evolution, with enhancements in highly epistatic traits being perceived as having diminished evolvability, the actual extent of pairwise inter‐gene epistasis remains a contentious topic. This is particularly true for heterologous pathways that incorporate genes from diverse species and thus, disparate evolutionary backgrounds. Following our evolutionary experiments, we conducted a combinatorial study to assess the prevalence of inter‐gene epistasis within heterologous pathway genes.

We evaluated the wild‐type TAL and its seven enhanced variants in two distinct contexts: one with 4CL and CHS in their wild‐type sequences, and another with their improved versions, 4CL‐11C1 and CHS‐9H9. As shown in Figure 2e, all seven enhanced TAL variants exhibited significantly lower naringenin production in the presence of wild‐type 4CL and CHS (4SI group). Conversely, the wild‐type TAL demonstrated marginally higher production when paired with the improved 4CL and CHS. Among all the TAL mutants, TAL‐26E7 or −28D11 showed strong sign epistasis with 4CL‐11C1 and CHS‐9H9 mutants, while the remaining TAL mutants displayed varying degrees of positive epistasis (Figure S9, Supporting Information). We also examined the improved 4CL and CHS mutants in similar contexts. 4CL‐11C1 exhibited a slight negative epistasis with TAL‐26E7 + CHS‐9H9, while CHS‐9H9 displayed reciprocal sign epistasis with TAL‐26E7 and 4CL‐11C1 (Figure 4c,e). Although our analyses were limited in scope, they underscored the presence of complex epistasis among heterologous pathway genes. These complex epistases significantly hindered the evolution of multiple enzymes in a given heterologous pathway, trapping the evolution outcome in a local maximum.^[^ ^4^, ^6^ ^]^ Furthermore, directed evolution with a randomized mutant library has often been described as “luck” or “chance”, as it is difficult, if not impossible, to predict the outcome of a given directed evolution task.^[^ ^4^, ^5^, ^8^ ^]^ Above phenomenon requires us to reduce the uncertainty of each directed evolution task by treating each pathway enzyme individually and creating an artificial bottleneck in the pathway to set the enzyme to be evolved in a well‐controlled evolutionary trajectory, where a clear lower‐bound of its evolutionary space was known to reduce the intrinsic randomness of the directed evolution process.^[^ ^6a^ ^]^ This also highlights the necessity for a method that can create predictable evolutionary trajectories, enabling the improvement of these pathways in a more controlled manner. The combination of three mutant enzymes resulted in higher naringenin producers, also showing the robustness of our methodology.

### 2.4 ProEnsemble Improves Naringenin Production by Promoter Engineering to Further Relax the Epistasis

With all the above‐mentioned epistases, we suspected that the directed evolution of three enzymes may further disrupt the metabolic flux. Therefore, we further optimised the expression of each pathway enzyme by promoter engineering with the aid of a machine learning algorithm ProEnsemble to improve the production of naringenin.^[^ ^3^, ^18^ ^]^ We first selected 42 reported promoters with a broad dynamic range from the literature and validated their strength using a mKate2 reporter^[^ ^19^ ^]^ (Figure S10, Supporting Information). Twelve promoters with varying strengths were then selected for downstream experiments, which were classified into three groups (high, medium and low strength). The strongest promoter was P_23104_, which exhibited a higher fluorescence than P_T7_ and P_BAD_ at 24 h, whereas the lowest fluorescence was observed for P_rrnA_, indicating a 211‐fold dynamic range for all the selected promoters (Figure S11, Supporting Information).

A previously reported Golden Gate method, which used mKate2 and ccdB as reporters to ensure high‐efficiency promoter and pathway assembly,^[^ ^20^ ^]^ was used to generate a random promoter library for the naringenin pathway. Of the 20 sequenced random clones, we achieved a 100% assembly success rate with a diverse set of promoters. As it required a sampling of more than 267000 clones to achieve a 95% probability to cover the whole library, which would be difficult to perform even with automation, we collected a subset of the library and used a machine learning algorithm to optimise the promoter combination. A previously reported Al^3+^ assay was used to screen for variants with higher naringenin titers.^[^ ^17^ ^]^ In this assay, the presence of Al^3+^ in a supernatant containing naringenin results in an absorption peak at 373 nm. This signal can be employed to approximate the concentration of naringenin. Notably, the signal ranges from 0.01 to 1.34 for naringenin concentrations between 0 and 1500 mg L^−1^ (Figure S1, Supporting Information). To avoid being trapped in a local maximum, we collected a balanced dataset with a focus on high naringenin production from about 1000 screened mutants using the Al^3+^assay. A total of 108 mutants with an Al^3+^ signal higher than 0.2 (corresponding to 130 mg L^−1^ Naringenin) were selected to represent high producers, whereas fifty samples with an Al^3+^ signal less than 0.2 were randomly picked from each plate to improve the generalizability of the model. In total, 158 mutants were selected as hits, and their naringenin titers were validated by HPLC, which ranged from 50.8 to 1044 mg L^−1^ (Extended Data Tabel S1, Supporting Information). The highest titer was produced by NAR1.0, which used P_1–29_ for TAL‐26E7, P_1–16_ for 4CL‐11C1, P_1–17_ for CHS‐9H9, and P_trxA_ for CHI, and it was 4.44‐fold higher than the control with three mutant enzymes under P_T7_. These results demonstrated the presence of an imbalanced metabolic flux and emphasized the importance of promoter optimization.

**Figure 5:**
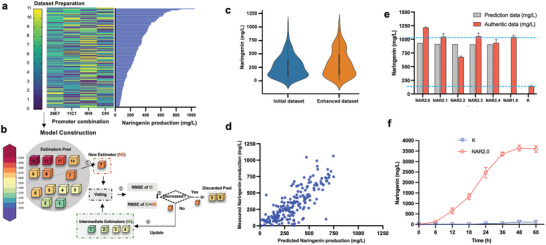
ProEnsemble improves naringenin production by optimizing the promoter combinations of the engineered pathway. a) Data collection for machine learning. 158 strains with diverse naringenin production were pre‐screened from ten 96‐deep wells using the Al^3+^ assay. The promoter combination and naringenin production were determined using Sanger sequencing and HPLC, respectively, and these two datasets were used to train the ProEnsemble model. b) Scheme of the ProEnsemble model. The Root Mean Square Errors (RMSEs) of 13 base estimators were evaluated based on a tenfold cross‐validation of the prepared dataset. All base estimators were then sorted by their RMSE from low to high, which were sequentially integrated into the model if the averaged naringenin prediction from the model with this specific estimator decreased the overall RMSE. The optimal model was the ensemble of gradient boosting regressor, ridge regressor, and gradient boosting. c) The initial and enhanced dataset for ProEnsemble model. There was an imbalance between the number of samples with high and low naringenin production in the initial dataset, which may decrease the ProEnsemble efficiency to accurately predict the promoter combinations of higher titers. Therefore, an enhanced dataset was collected to solve this issue. d) The Pearson's Correlation Coefficient (PCC) of the measured naringenin concentrations versus the predicted values by the optimal ProEnsemble model. e) Naringenin productions in diverse strains. NAR2.X strain, the top five strains as predicted by ProEnsemble model. NAR1.0, the strain with the highest naringenin production from the initial dataset. K, the strain with the naringenin‐associated genes under the control of P_T7_. Naringenin concentrations were determined by HPLC in biological triplicates and the error bars represent the standard deviation. f) The fed‐batch fermentation of NAR2.0 and K strains. K, the strain with the naringenin‐associated genes under the control of P_T7_. NAR2.0, the strain with the highest naringenin production in this study. Naringenin concentrations were determined by HPLC from three independent fed‐batch fermentations and the error bars represent the standard deviation.

Next, we proposed a promoter combinations prediction framework called ProEnsemble, which is based on Ensemble models^[^ ^21^ ^]^ (**Figure** **5**). ProEnsemble was designed with representative base estimators, and their prediction results were integrated for better accuracy. Specifically, we evaluated the Root Mean Square Error (RMSE) of 13 base estimators based on the tenfold cross‐validation of the abovementioned dataset with 158 mutants (Figure 5a,b). All base estimators were then sorted by their RMSE from low to high. Any base estimator that yielded an averaged naringenin prediction with decreased overall RMSE was integrated into the model. The optimal model is the ensemble of gradient boosting regressor, ridge regressor, gradient boosting with categorical features regressor, lasso regressor and extreme gradient boosting regressor, which showed a minimum RMSE of 135 (Data Tabel S2, Supporting Information). The Pearson's Correlation Coefficient (PCC) also demonstrated a better correlation between the experimentally measured values and predicted values with the optimised model.

Five top hits from the ProEnsemble model prediction all produced a naringenin titer above 700 mg L^−1^. In contrast, only five out of 960 random samples from the first‐round analysis exhibited similar activity, highlighting the effectiveness of the model. However, none of the hits outperformed the top strain NAR1.0 (Data Tabel S3, Supporting Information). An imbalance was observed in the distribution of samples with high and low naringenin production levels, which may hinder the model's accuracy in predicting promoter combinations for higher naringenin production (Figure 5c). Only 26% of the library showed naringenin production above 400 mg L^−1^. To address this issue, 68 additional samples with an Al^3+^signal above 0.3 were collected from another 1500 clones (Data Tabel S4, Supporting Information). We further optimised the model by expanding the training set with the inclusion of results with naringenin titers higher than 400, 500, 600, 700 and 800 mg L^−1^, respectively. The best performance was achieved by adding 27 data points above 600 mg L^−1^ to the initial dataset, which was even better than when all the data points were added (Data Tabel S2, Supporting Information). The modified dataset slightly increased the RMSE by 5.16% compared to the initial dataset, while the PCC was improved from 74% to 82%, showing the importance of a balanced sample distribution to the model performance (Figure 5d). Five top hits predicted by the improved model all showed high naringenin production, with the highest titer of 1.21 g L^−1^ for NAR2.0, which was 16% higher than that of strain NAR1.0 and 5.16‐fold higher than that of the initial constructs without promoter optimization (Figure 5e). It is noting that more than 99.11% of the strains in the random promoter libraries produced lower naringenin titers than 1 g L^−1^, suggesting ProEnsemble could significantly improve our chance of discovering better hits. The titer of NAR2.0 was also 5.92‐fold higher than that of strain WT2.0 (Figure S13, Supporting Information), revealing that pathway evolution from the above bottlenecking‐debottlenecking strategy is beneficial for naringenin production.

Using the optimum promoter combination, fed‐batch fermentation was performed in a 1‐L fermenter. As shown in Figure 5f, the wild‐type enzymes without promoter optimisation produced an undetectable level of naringenin at 12 h and only 116 mg L^−1^ naringenin at 48 hours, while the mutants with optimised promoters reached 660 mg L^−1^ in 12 hours and reached a peak at 3.65 g L^−1^ at 48 h (Figure 5f). To our knowledge, this is the highest titer in the literature for naringenin production directly from tyrosine, which is 3.41‐fold compared to the previous record of directly from tyrosine and 3.02‐fold compared to the previous record of fermentation with coumaric acid as intermediate feeding.^[^ ^22^ ^]^ Given that only the pathway enzymes and promoters were modified in this work, future metabolic engineering has the potential to further increase the naringenin titer.

### 2.5 Demonstration as a General Flavonoid Chassis

**Figure 6:**
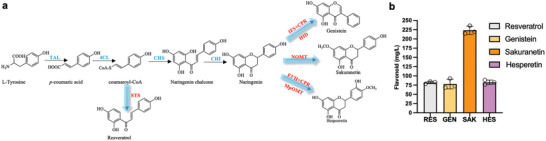
Production of other flavonoids using the naringenin host. (a) Pathways of the candidate flavonoids, including resveratrol, genistein and sakuranetin. Tyrosine can be converted to resveratrol by three genes, including tyrosine ammonia‐lyase (TAL), 4‐coumarate‐CoA ligase (4CL) and stilbene synthase (STS). Genistein can be biosynthesized from naringenin by overexpressing 2‐hydroxyisoflavanone synthase (IFS), cytochrome P450 enzyme reductase (CPR) and 2‐hydroxyisoflavanone dehydratase (HID). Sakuranetin can be produced by overexpressing O‐methyltransferase, NOMT. Naringenin is converted to hesperetin by overexpressing flavonoid 3′‐hydroxylase (F3′H), cytochrome P450 reductase (CPR) and O‐methyltransferase (MpOMT). (b) Flavonoid production in diverse strains. RES, resveratrol biosynthesis strain; GEN, genistein biosynthesis strain; SAK, sakuranetin biosynthesis strain; HES, hesperetin biosynthesis strain. The concentrations of each target compounds were determined by HPLC in biological triplicates and the error bars represent the standard deviation.

We further demonstrated that the optimised constructs could serve as a versatile flavonoid chassis by producing four different flavonoids via different intermediates in the naringenin pathway, including resveratrol from coumaroyl‐CoA and genistein, sakuranetin, and hesperetin from naringenin (**Figure** **6**; Table S11, Supporting Information). The same constructs used in previous literature were used for a fair comparison, although multiple additional strategies were also utilized in those studies. To produce resveratrol, the stilbene synthase from *Vitis vinifera* (VvSTS) under the control of P_T7_ in the pETduet‐1 vector was transformed into competent cells with TAL‐26E7 and 4CL‐11C1 to complete its biosynthetic pathway (Note S4, Supporting Information). Without any other metabolic engineering, the resulting strain produced 82.1 mg L^−1^ resveratrol, significantly higher than the 35.0 mg L^−1^ observed from a previous resveratrol producer with an engineered malonyl‐CoA supply from malonate,^[^ ^23^ ^]^ and comparable to the 80.4 mg L^−1^ produced by a previous resveratrol producer with an engineered tyrosine supply module.^[^ ^24^ ^]^ Similarly, 2‐hydroxyisoflavanone synthase from *Lotus japonicus* KKK‐LjtIFS, 2‐hydroxyisoflavanone dehydratase from *Glycine max* GmHID and cytochrome P450 reductase from *Lotus japonicus* OmpAL‐LjtCPR were introduced to the NAR2.0 strain according to the literature for genistein production.^[^ ^25^ ^]^ O‐methyltransferase NOMT from *Oryza sativa* L. cv. Nakdong was introduced into the NAR2.0 strain to obtain a sakuranetin producer.^[^ ^26^ ^]^ Flavonoid 3′‐hydroxylase (F3'H) from *Gentiana triflora*, cytochrome P450 reductase (CPR) from *Arabidopsis thaliana* and O‐methyltransferase MpOMT from *Mentha piperita* were overexpressed for hesperetin biosynthesis.^[^ ^27^ ^]^ The resulting strains generated 77.9 mg L^−1^ genistein, 223 mg L^−1^ sakuranetin and 82.5 mg L^−1^ hesperetin, which were 1.28‐fold, 1.29‐fold, and 2.22‐fold higher, respectively, than the highest titers in the literature, which employed more complicated engineering steps and co‐culture strategies (Table S11, Supporting Information).

## 3 Conclusion

In summary, we have investigated the epistasis among heterologous pathway genes and proposed a clear evolutionary trajectory for pathway evolution at a controllable space. We have further demonstrated the feasibility of a strategy combining biofoundry‐assisted pathway bottlenecking and debottlenecking to separate the target enzyme from other factors which may interfere with its evolution, followed by machine learning‐based promoter optimization to significantly enhance pathway flux. By utilizing this strategy, a parallel evolution of all naringenin pathway enzymes along a predictable evolutionary trajectory in six weeks, whose epistasis was further relaxed by ProEnsemble algorithm, leading to a final naringenin titer of 1.21 g L^−1^ in 96‐deep well plates and 3.65 g L^−1^ in fed‐batch fermentation. The titer surpassed all previously reported results in the literature. We have also demonstrated the potential of this optimal strain as an effective chassis for flavonoids production. This approach can be readily adapted for any given metabolic pathway, paving the way for automated chassis construction in contemporary biofoundries.

## 4 Experimental Section

### Strains and Cultivation Conditions

The plasmids and strains used in this study are summarized in Tables S1 and S2 (Supporting Information). *Escherichia coli* DH5α was chosen as the host for plasmid construction. Antibiotics (50 µg mL^−1^ streptomycin and 25 µg mL^−1^ chloramphenicol) were used for plasmid maintenance. *E. coli* BL21(DE3) was used for gene expression in MOPS (3‐(N‐morpholino)propanesulfonic acid) medium as described previously.^[^ ^17^, ^28^ ^]^ Luria‐Bertani (LB; 10 g L^−1^ tryptone, 10 g L^−1^ NaCl, and 5 g L^−1^ yeast extract) medium was used for strain maintenance and seed propagation. All chemicals were reagent grade and purchased from Sigma–Aldrich (St. Louis, MO, USA). Molecular manipulation‐associated reagents, such as T4 ligase and BsaI, were purchased from NEB (Beverly, MA, USA).

### Parallel Evolution of all Pathway Enzymes by Automation

Error‐prone PCR was used to generate the mutation libraries using a GeneMorph II Random Mutagenesis Kit (Agilent Technologies, Santa Clara, CA, USA). The low mutation frequency (0‐5 mutations/kb) was obtained by regulating the initial target gene amounts (100 ng) and the fewer cycle numbers (25 cycles). Phata polymerase (Nanjing, Jiangsu, China) was used to linearize the pBbS8C plasmids. Based on the homology between the error‐prone PCR products and the linearized pBbS8C, the mutation libraries were constructed by Gibson assembly. The individual mutation libraries were further transformed into the relevant competent cells and spread on LB agar plates (Table S1, Supporting Information).

The automated workstation comprised 14 pieces of equipment (Figure S3, Supporting Information), where the spinnaker and liquid handler system were connected to diverse devices, including an incubator, centrifuge, and microplate reader. This automated workstation was used to screen the mutation library (Figures S4–S7 and Video S1–S4, Supporting Information). In detail, the mutants from LB agar plates were automatically distinguished using QPix 400 based on the sizes, roundness, and distances with the adjacent stains. The QPix 400 system was then employed to pick mutant clones (≈5000 clones for the individual evolving gene) into the 96‐deep well plates with 0.8 mL LB medium (Figure S4 and Video S1, Supporting Information). The resulting 96‐deep well plates were incubated at 800 rpm and 30 °C overnight. Then, an aliquot of the culture was inoculated (1% v/v) into 96‐deep well plates with 0.8 mL MOPS medium, and the plates were incubated at 800 rpm and 30 °C for 2 days (Figure S5 and Video S2, Supporting Information). After this, the 96‐deep well plates were centrifuged at 5 000 rpm for 10 min (Figure S6 and Video S3, Supporting Information). The naringenin content of the cultures in the 96‐well plates was evaluated using an Al^3+^assay (Figures S2 and S6 and Video S3, Supporting Information).^[^ ^17^ ^]^ The Momentum software ensured that the computer automatically calculated the naringenin content of the individual 96‐deep wells based on a spectrophotometry assay of Al^3+^ absorption. Then, the computer marked the candidate mutants, whose Al^3+^ signal thresholds were higher than that of the corresponding control data of the TAL (0.15), 4CL (0.08), and CHS (0.05) mutation libraries. Then, the liquid handler system resuspended the marked hits and transferred 300 µL per well of fermentation broth into the new 96‐deep well plates (Figure S7 and Video S4, Supporting Information). An equal volume of ethanol was added into the individual wells, and the plates were vortexed twice at 800 rpm for 1 min each. The mixture was placed at room temperature for 30 min and centrifuged at 5,000 rpm for 10 min. Approximately 500 µL of supernatant was used for HPLC validation (Figure S7 and Video S4, Supporting Information). Those mutants with higher titers by HPLC were chosen for the following experiments and sequenced.

### HPLC Methods for Naringenin Detection

Naringenin titers were detected at 290 nm using an Agilent 1260 HPLC system (Waldbronn, Germany) equipped with a diode array detector (DAD) 1260 model VL + (G7115A) and a C18 column (3 × 100 mm 2.7 µm). The column was eluted with gradient elution at 30°C and 0.3 mL min^−1^ flow rate: 10% to 40% acetonitrile/water (vol/vol) for 5 min, 40% acetonitrile (vol/vol) for 7 min, 40% to 95% acetonitrile (vol/vol) for 3 min, and 95% to 10% acetonitrile (vol/vol) for 3 min. It was noted that 0.3% acetic acid (vol/vol) was added to above mobile phases, including the acetonitrile or water, which contributed to naringenin separation.

### Determination of Enzymatic Kinetic Parameters

The clones of wild‐type genes and mutants were incubated at 37 °C overnight and 200 rpm in LB media. About 1% inoculation dose of different samples was transferred into 250 mL shake flasks with 100 mL LB media. Isopropyl β‐D‐Thiogalactoside (1 mm) was added into flasks until the strains reached OD_600_ of 0.6. These strains were further incubated for 10 h at 16 °C and 100 rpm. Then, the inducible strains were lysed by ultrasonication. The purified proteins were obtained by the standard protocol of BeaverBeads IDA‐Nickel (Beaver, Boston, USA).

Enzyme kinetic parameters of TAL and the mutant were determined using the method from *Zhou* et al.^[^ ^15^ ^]^ In detail, the wild‐type TAL and mutants were tested in a 200 µL reaction volume. The purified protein (1 µg) was added into reactions comprising 90 µL Tris‐HCl buffer (50 mm, pH 8.5) and different concentrations of L‐tyrosine. The mixture was cultured at 40 °C for 30 min and monitored for the appearance of coumaric acid at 315 nm.^[^ ^15^ ^]^ One unit of enzyme activity is defined as 1 µm *p*‐coumaric acid production in one minute.

Enzymatic kinetic parameters of 4CL and the mutant were determined using the method from Alberstein et al.^[^ ^29^ ^]^ The reaction system comprised the purified protein (1 µg), 5 mm ATP, 5 mm MgSO_4_, 5 mm CoA, and different concentrations of *p*‐coumaric acid in Tris‐HCl buffer (0.4 m, pH 7.8). The mixture without CoA was chosen as the control. The reaction was cultured at 30 °C. The production of *p*‐coumaroyl CoA was determined at 333 nm.

Enzymatic kinetic parameters of CHS and the mutant were determined using the method from Kong et al. with some modification.^[^ ^30^ ^]^ The reaction system contained the purified protein (1 µg), 200 µm malonyl‐CoA, and different concentrations of *p*‐coumaroyl‐CoA in potassium phosphate buffer (0.1 m, pH 7.0). The reaction mixture was cultured at 30°C and 450 rpm for 4 h in the dark. Then an equal volume of ethanol was added to the mixture. Naringenin chalcone was determined using the HPLC method described above. Kinetic parameters of different proteins, including *K_m_*, V*_max,_* and *K_cat_*/*K_m_*, were calculated using Lineweaver‐Burk plots.

### Further Relax the Epistasis of the Evolved Pathway Using ProEnsemble Framework‐Mediated Promoter Engineering

Promoters with different expression strengths were used to optimise the engineered naringenin pathway. To simplify the promoter choice, those promoters were screened that had been previously reported.^[^ ^19^ ^]^ these promoters were cloned into pBbS8C‐mKate2 (Note S2, Supporting Information) and transformed them into BL21(DE3). Diverse strains were picked into 96‐deep wells with 800 µL of LB media and cultured at 30 °C overnight. Then 8 µL fermentation broths were transferred into 96‐deep wells with 0.8 mL MOPS media and incubated at 30 °C. Fluorescence signals of the mKate2 protein were measured using excitation and emission filters of 588 and 633 nm, respectively.^[^ ^31^ ^]^ Optical density was tested at 600 nm to evaluate the strain growth status. The P_T7_ and P_BAD_ promoters were chosen as the control promoters. Based on the fluorescence strength variances, 12 promoters were screened and divided them into three types, including weak, medium, and strong promoters (Figures S9 and S10, Supporting Information).

Promoter engineering was further employed to balance the engineered naringenin pathway. HamediRad and coworkers had determined that linkers with higher affinity and specificity can contribute to the efficiency of Golden Gate assembly.^[^ ^32^ ^]^ Therefore, five 4‐bp linkers (ATCT, GCTG, CGCT, TCAT, and GAGT) were used to ensure a higher efficient promoter library (Note S3, Supporting Information).^[^ ^32^ ^]^ Next, the backbone plasmid was equipped with mKate2 and ccdB proteins. The ccdB protein could lock up DNA gyrase by damaging double‐stranded DNA, which ultimately triggers the death of negative clones with the backbone plasmid.^[^ ^33^ ^]^ Thus, the amounts of clones with ccdB protein grew on the plates, whereas no red morphology of the negative clones was found (Note S3, Supporting Information). Then, the promoter library was prepared by mixing the candidate plasmids in equal proportions. Strains with diverse naringenin productions were prescreened from ten 96‐deep wells of the promoter library based on the differences in their Al^3+^ assay.^[^ ^17^ ^]^ The actual naringenin production was determined using the above HPLC method.

In ProEnsemble, the goal was to establish the relationship between diverse promoter combinations and naringenin production. 12 different types of promoters were encoded using one‐hot encoding (Table S7, Supporting Information). The corresponding label was naringenin production, which was a continuous numerical value from tens to thousands. For greater prediction performance, as many base estimators as possible were selected. Specifically, 13 representative models ranging from simple linear models to complicated ensemble models were chosen, containing ridge regressor, lasso regressor, k‐neighbors regressor, support vector regressor, decision tree regressor, random forest regressor, extra trees regressor, adaptive boosting regressor, bootstrap aggregating regressor, gradient boosting regressor, extreme gradient boosting regressor, light gradient boosting regressor, and gradient boosting with categorical features regressor. The Root Mean Square Error (RMSE) of 13 base estimators based on a tenfold cross‐validation of the initial dataset was compared first. For better integration of these base estimators, which placed in descending order by error. The base estimators were successively added to the ensemble model if the error was reduced. The final prediction value of naringenin production was averaged using the selected models. Additionally, RMSE and Pearson Correlation Coefficient (PCC) were selected as evaluation metrics in Equations (1) and (2), where *y_ie_* denotes the experimentally measured naringenin production, *y_ip_* denotes the predicted naringenin production, ${\bar{y}}_{e}$ denotes the average of the naringenin production, ${\bar{y}}_{p}$ denotes the average of the predicted naringenin production and n denotes the number of samples. The link of ProEnsemble framework is placed at the corresponding website (https://github.com/Luo‐SynBioLab/ProEnsemble).

(1) $$PCC=\frac{1}{n}\frac{\sum_{i=1}^{n}\left(y_{ie}-{\bar{y}}_{e}\right)\left(y_{ip}-{\bar{y}}_{p}\right)}{\sqrt{\sum_{i=1}^{n}{\left(y_{ie}-{\bar{y}}_{e}\right)}^{2}}\sqrt{\sum_{i=1}^{n}{\left(y_{ip}-{\bar{y}}_{p}\right)}^{2}}}$$

(2) $$RMSE=\sqrt{\frac{\sum_{i=1}^{n}{\left(y_{ie}-y_{ip}\right)}^{2}}{n}}$$

### Bioreactor Production

To further evaluate the naringenin production of the engineered strains, a batch bioreactor experiment was carried out in a DASGIP MX4/4 bioreactor system (Eppendorf, Hamburg, Germany). Before incubation, the process parameters were set at 400 rpm, 30 °C, and pH 7.0. The zero signal of the pO_2_ electrode was calibrated at 0%. In contrast, a stirrer speed of 1200 rpm was used as the 100% level of pO_2_ electrode. About 1 mL of the antifoaming agent was added. The bioreactor production was performed using the following process. A single clone was resuspended in a 500 mL flask with 100 mL LB media and cultured overnight at 200 rpm and 30 °C. The fermentation broth was centrifuged when the strains reached the logarithmic growth phase. The supernatant was discarded, and the strains were resuspended in MOPS media and transferred to the bioreactor. The temperature was kept at 30 °C. The pH was automatically maintained at 7.0 by the addition of 2 m ammonium hydroxide and 0.5 M H_2_SO_4_. Samples of approximately 5 mL were regularly removed to evaluate the growth status and metabolite production.

## Conflict of Interest

X.Luo has financial interests in Demetrix and Synceres. J.D.K. has financial interests in Amyris, Ansa Biotechnologies, Apertor Pharma, Berkeley Yeast, Cyklos Materials, Demetrix, Lygos, Napigen, ResVita Bio, Zero Acre Farms and BioMia.

## Author Contributions

H.D. and H.Y. contributed equally to this work. Conceptualization performed by J.D.K. and X.L. Data collection performed by H.D., H.Y., Y.D., Y.Q., F.L., X.W., J.H., W.L., Y.L., L.Q., and Z.Z. Genetic analysis was done by H.D., Y.D., and W.L. Statistical analysis performed by H.Y., F.L., Y.Z., and J.H. Visualization performed by H.D., Y.Z., and H.Y. Methodology performed by H.D., H.Y., Y.D., and J.H. Supervision was performed by J.D.K. and X.L. Writing—original draft validated by H.D. and X.L. Writing—review and editing were done by H.D., H.Y., Y.D., Y.Z., J.H., J.D.K., and X.L.

## Supporting information

Supporting Information

Supplemental Data1

Supplemental Data2

Supplemental Data3

Supplemental Data4

Supplemental Data5

Supplemental Table1

Supplemental Table2

Supplemental Table3

Supplemental Table4

Supplemental Table5

Supplemental Video1

Supplemental Video2

Supplemental Video3

Supplemental Video4

## Data Availability

The data that support the findings of this study are available in the supplementary material of this article.
