# Supplementary material for: Pathway Evolution Through a Bottlenecking‐Debottlenecking Strategy and Machine Learning‐Aided Flux Balancing
Source: Adv Sci (Weinh). 2024 Feb 6;11(14):2306935. doi: 10.1002/advs.202306935 (PMC11005738; doi:10.1002/advs.202306935)
Supplement: Supplementary file 1 — Supporting Information [file ADVS-11-2306935-s009.pdf]

ADVANCED SCIENCE
Open Access

Supporting Information

for *Adv. Sci.*, DOI 10.1002/advs.202306935

Pathway Evolution Through a Bottlenecking-Debottlenecking Strategy and Machine Learning-Aided Flux Balancing

Huaxiang Deng, Han Yu, Yanwu Deng, Yulan Qiu, Feifei Li, Xinran Wang, Jiahui He, Weiyue Liang, Yunquan Lan, Longjiang Qiao, Zhiyu Zhang, Yunfeng Zhang, Jay D. Keasling and Xiaozhou Luo**

Supporting Information

Pathway evolution through a bottlenecking-debottlenecking strategy and machine learning-aided flux balancing

Huaxiang Deng[†], *Han Yu*[†], *Yanwu Deng , Yulan Qiu, Feifei Li, Xinran Wang, Jiahui He, Weiyue Liang, Yunquan Lan, Longjiang Qiao, Zhiyu Zhang, Jay D. Keasling*, Xiaozhou Luo**

Table of Contents

Supplementary Notes 3

Supplementary Figures 6

Supplementary Tables 23

Supporting References 38

Supplementary Notes

Supplementary Notes 1. Detailed description on the constructing plasmids and strains in Figure 1.

To create artificial bottlenecks for the directed evolution of enzymes in the naringenin pathway, we need to construct a simple plasmid for naringenin production. Before that, suitable naringenin-associated genes, including TAL, 4CL, CHS, and CHI, were required because combinations of naringenin-associated genes from diverse species sources could influence naringenin titers[9]. Previous work has proved that the synergetic cooperation of four optimal genes in the pCDF plasmid can generate higher naringenin production[10]. Thus, we chose this optimal gene combination as the original plasmid for naringenin biosynthesis.

Then, four genes from the pCDF plasmid were cloned into pBbS8C, pBba8C and pBbb8a plasmids by Gibson assembly, respectively. The element, ranging from araC gene to TAL gene, was amplified from pBbS8C-TAL. This element was further cloned into pBbE5K and RSF plasmids, resulting in pBbE5K-pBAD-TAL and RSF-pBAD-TAL. Genes, including 4CL, CHS, and CHI, were also cloned into pBbE5K and RSF plasmid as above similar process.

To search the artificial naringenin bottleneck pathway, diverse plasmids with diverse TAL copy numbers were transformed into the competent cells with the CDF-4SI plasmid. Diverse plasmids with diverse 4CL copy numbers were transformed into the competent cells with the CDF-TSI plasmid. Diverse plasmids with diverse CHS copy numbers were transformed into the competent cells with the CDF-T4I plasmid. Diverse plasmids with diverse CHI copy numbers were transformed into the competent cells with the CDF-T4S plasmid.

Supplementary Notes 2. Relevant information on confirming expression strength levels.

(1) Sequence information of the pBbS8C-mKate2-ccdB plasmid.

RBS sequences were underlined. Therein, these expression levels of these RBS sequences were confirmed by previously[7]. The superfolder GFP (sfGFP) was marked as green and used to confirm whether promoters are bidirectional expression. The mKate2 protein was marked as red and used to determine expression strength levels of diverse promoters. YFP was marked as yellow and utilized to evaluate whether the BioFab terminator FAB391 can stop mKate2 expression. The diverse promoters were cloned into the NNNNNN sites, which were marked as purple. Therein, the BioFab terminator FAB391 was marked as bold.

gacgtcttatgacaacttgacggctacatcattcactttttcttcacaaccggcacggaactcgctcgggctggccccggtgcattttttaaatacccgcgagaaatagagttgatcgtcaaaaccaacattgcgaccgacg
gtggcgataggcatccgggtggtgctcaaaagcagcttcgcctggctgatacgttggtcctcgcgccagcttaagacgctaatccctaactgctggcggaaaagatgtgacagacgcgacggcgacaagcaaacatg
ctgtgcgacgctggcgatatcaaaattgctgtctgccaggtgatcgctgatgtactgacaagcctcgcgtacccgattatccatcggtggatggagcgactcgttaatcgcttccatgcgccgcagtaacaattgctcaag
cagatttatcgccagcagctccgaatagcgcccttccccttgcccggcgttaatgatttgcccaaacaggtcgctgaaatgcggctggtgcgcttcatccgggcgaaagaaccccgtattggcaaatattgacggccagt
taagccattcatgccagtaggcgcgcggacgaaagtaaacccactggtgataccattcgcgagcctccggatgacgaccgtagtgatgaatctctcctggcgggaacagcaaaatatcacccggtcggcaaacaaat
tctcgtccctgatttttcaccaccccctgaccgcgaatggtgagattgagaatataacctttcattcccagcggtcggtcgataaaaaaatcgagataaccgttggcctcaatcggcgttaaacccgccaccagatgggca
ttaaacgagtatcccggcagcaggggatcattttgcgcttcagccatacttttcatactcccgccattcagagaagaaaccaattgtccatattgcatcagacattgccgtcactgcgtcttttactggctcttctcgctaacca
aaccggtaaccccgcttattaaaagcattctgtaacaaagcgggaccaaagccatgacaaaaacgcgtaacaaaagtgtctataatcacggcagaaaagtccacattgattatttgcacggcgtcacactttgctatgcc
atagcatttttatccataagattagcggattctacctgacgctttttatcgcaactctctactgtttctccatacccgtttttttgggaattcggatccaaactcgagtaaggatctccaggcatcaaataaaacgaaaggctcagt
cgaaagactgggcctttcgttttatctgttgtttgtcggtgaacgctctctactagagtcacactggctcaccttcgggtgggcctttctgcgtttatacctaggtcatttgtacagttcatccataccatgcgtgatgcccgctg
cggttacgaactccagcagaaccatatgatcgcgtttctcgttcggatctttagacagaacgctttgcgtgctcagatagtgattgtctggcagcagaacaggaccatcaccgattggagtgttttgctggtagtgatcagc
cagctgcacgctgccatcctccacgttgtggcgaattttaaaattcgctttaatgccatttttttgtttatcggcggtgatgtaaacattgtggctgttaaaattgtattccagcttatggcccaggatattgccgtcttctttaaagt
caatgcctttcagctcaatgcggtttaccagggtatcgccttcaaatttcacttccgcacgcgttttgtacgtgccgtcatccttaaaggaaatcgtgcgttcctgcacatagccttccggcatggcggacttgaagaagtcat
gctgcttcatatggtccggataacgagcaaagcactgaacaccataagtcagcgtcgttaccagagtcggccaaggtaccggcagtttaccagtagtacagatgaacttcagcgtcagtttaccattagttgcgtcacctt
caccctcgccacgcacggaaaacttatgaccgttgacatcaccatccagttccaccagaatagggacgacaccagtgaacagctcttcgcctttacgcatGTCTGTCGTGGTAAAAGCGTTAAG
GAGTGTTTTGG**NNNNNN**ccatcttagtatattagttaagtataagaaggagatataATGGTTAGCGAGCTGATCAAAGAAAACATGCACATGAAACTGTATA
TGGAAGGCACCGTGAATAACCACCACTTTAAATGTACCAGCGAAGGTGAAGGTAAACCGTATGAAGGCACCCAGACCATGXCGTA
TTAAAGCAGTTGAAGGTGGTCCGCTGCCGTTTGCATTTGATATTCTGGCAACCAGCTTTATGTATGGCAGCAAAACCTTTATTAACC
ATACCCAGGGTATCCCGGATTTTTTCAAACAGAGCTTTCCGGAAGGTTTTACCTGGGAACGTGTTACCACCTATGAAGATGGTGGT
GTTCTGACCGCAACCCAGGATACCAGTCTGCAGGATGGTTGTCTGATTTATAATGTGAAAATTCGCGGTGTGAACTTTCCGAGCAA
TGGTCCGGTTATGCAGAAAAAAACCCTGGGTTGGGAAGCAAGCACCGAAACCCTGTATCCGGCAGATGGTGGTCTGGAAGGTCGT
GCAGATATGGCACTGAAACTGGTTGGTGGTGGTCATCTGATTTGCAATCTGAAAACCACCTATCGTAGCAAAAAACCGGCAAAAA
ATCTGAAAATGCCTGGCGTGTATTATGTTGATCGTCGTCTGGAACGTATTAAAGAGGCAGATAAAGAAACCTATGTGGAACAGCAT
GAAGTTGCAGTTGCACGTTATTGTGATCTGCCGAGCAAACTGGGTCACCGCTGATAACCATGGGCTAGCGGTTTGAAGGGTATTGG
**TCGGTCAGTTTCACCTGATTTACGTAAAAACCCGCTTCGGCGGGTTTTTGCTTTTGGAGGGGCAGAAAGATGAATGACTGT
C**GTTTATAATTTAAAAGCCAATTATAAATCAGTCATGCGTAAAGGAGAAGAACTTTTCACTGGAGTTGTCCCAATTCTTGTTGAATT
AGATGGTGATGTTAATGGGCACAAATTTTCTGTCAGTGGAGAGGGTGAAGGTGATGCAACATACGGAAAACTTACCCTTAAATTTA
TTTGCACTACTGGAAAACTACCTGTTCCATGGCCAACACTTGTCACTACTTTCGGTTATGGTCTAAAATGCTTTGCTAGATACCCAG
ATCATATGAAACGGCATGACTTTTTCAAGAGTGCCATGCCCGAAGGTTATGTACAGGAAAGAACTATATTTTTCAAAGATGACGGG
AACTACAAGACACGTGCTGAAGTCAAGTTTGAAGGTGATACCCTTGTTAATAGAATCGAGTTAAAAGGTATTGATTTTAAAGAAGA
TGGAAACATTCTTGGACACAAATTGGAATACAACTATAACTCACACAATGTATACATCATGGCAGACAAACAAAAGAATGGAATC
AAAGTTAACTTCAAAATTAGACACAACATTGAAGATGGAAGCGTTCAACTAGCAGACCATTATCAACAAAATACTCCAATTGGCG
ATGGCCCTGTCCTTTTACCAGACAACCATTACCTGTCCTATCAATCTGCCCTTTCGAAAGATCCCAACGAAAAGAGAGACCACATG
GTCCTTCTTGAGTTTGTAACAGCTGCTGGGATTACACATGGCATGGATGAACTATACAAATAAagaaatcatccttagcgaaagctaaggattttttttatctg
gtacgggttttgctgcccgcaaacgggctgttctggtgttgctagtttgttatcagaatcgcagatccggcttcagccggtttgccggctgaaagcgctatttcttccagaattgccatgatttttccccacgggaggcgtca
ctggctcccgtgttgtcggcagctttgattcgataagcagcatcgcctgtttcaggctgtctatgtgtgactgttgagctgtaacaagttgtctcaggtgttcaatttcatgttctagttgctttgttttactggtttcacctgttctatt
aggtgttacatgctgttcatctgttacattgtcgatctgttcatggtgaacagctttgaatgcaccaaaaactcgtaaaagctctgatgtatctatcttttttacaccgttttcatctgtgcatatggacagttttccctttgatatgtaa
cggtgaacagttgttctacttttgtttgttagtcttgatgcttcactgatagatacaagagccataagaacctcagatccttccgtatttagccagtatgttctctagtgtggttcgttgtttttgcgtgagccatgagaacgaacca
ttgagatcatacttactttgcatgtcactcaaaaattttgcctcaaaactggtgagctgaatttttgcagttaaagcatcgtgtagtgtttttcttagtccgttatgtaggtaggaatctgatgtaatggttgttggtattttgtcaccat
tcatttttatctggttgttctcaagttcggttacgagatccatttgtctatctagttcaacttggaaaatcaacgtatcagtcgggcggcctcgcttatcaaccaccaatttcatattgctgtaagtgtttaaatctttacttattggtttc
aaaacccattggttaagccttttaaactcatggtagttattttcaagcattaacatgaacttaaattcatcaaggctaatctctatatttgccttgtgagttttcttttgtgttagttcttttaataaccactcataaatcctcatagagtat
ttgttttcaaaagacttaacatgttccagattatattttatgaatttttttaactggaaaagataaggcaatatctcttcactaaaaactaattctaatttttcgcttgagaacttggcatagtttgtccactggaaaatctcaaagccttt
aaccaaaggattcctgatttccacagttctcgtcatcagctctctggttgctttagctaatacaccataagcattttccctactgatgttcatcatctgagcgtattggttataagtgaacgataccgtccgttctttccttgtagggt
tttcaatcgtggggttgagtagtgccacacagcataaaattagcttggtttcatgctccgttaagtcatagcgactaatcgctagttcatttgctttgaaaacaactaattcagacatacatctcaattggtctaggtgattttaatc
actataccaattgagatgggctagtcaatgataattactagtccttttcccgggtgatctgggtatctgtaaattctgctagacctttgctggaaaacttgtaaattctgctagaccctctgtaaattccgctagacctttgtgtgttt
tttttgtttatattcaagtggttataatttatagaataaagaaagaataaaaaaagataaaaagaatagatcccagccctgtgtataactcactactttagtcagttccgcagtattacaaaaggatgtcgcaaacgctgtttgctc
ctctacaaaacagaccttaaaaccctaaaggcttaagtagcaccctcgcaagctcgggcaaatcgctgaatattccttttgtctccgaccatcaggcacctgagtcgctgtctttttcgtgacattcagttcgctgcgctcac
ggctctggcagtgaatgggggtaaatggcactacaggcgccttttatggattcatgcaaggaaactacccataatacaagaaaagcccgtcacgggcttctcagggcgttttatggcgggtctgctatgtggtgctatctg
actttttgctgttcagcagttcctgccctctgattttccagtctgaccacttcggattatcccgtgacaggtcattcagactggctaatgcacccagtaaggcagcggtatcatcaacaggcttacccgtcttactgtccctagt
gcttggattctcaccaataaaaaacgcccggcggcaaccgagcgttctgaacaaatccagatggagttctgaggtcattactggatctatcaacaggagtccaagcgagctcgatatcaaattacgccccgccctgcca
ctcatcgcagtactgttgtaattcattaagcattctgccgacatggaagccatcacaaacggcatgatgaacctgaatcgccagcggcatcagcaccttgtcgccttgcgtataatatttgcccatggtgaaaacgggggc
gaagaagttgtccatattggccacgtttaaatcaaaactggtgaaactcacccagggattggctgagacgaaaaacatattctcaataaaccctttagggaaataggccaggttttcaccgtaacacgccacatcttgcga

atatatgtgtagaaactgccggaaatcgtcgtggtattcactccagagcgatgaaaacgtttcagtttgctcatggaaaacggtgtaacaagggtgaacactatcccatatcaccagctcaccgtctttcattgccatacgaa
attccggatgagcattcatcaggcgggcaagaatgtgaataaaggccggataaaacttgtgcttatttttctttacggtctttaaaaaggccgtaatatccagctgaacggtctggttataggtacattgagcaactgactga
aatgcctcaaaatgttctttacgatgccattgggatatatcaacggtggtatatccagtgatttttttctccattttagcttccttagctcctgaaaatctcgataactcaaaaaatacgcccggtagtgatcttatttcattatggtga
aagttggaacctcttacgtgccgatcaacgtctcattttcgccagatatc

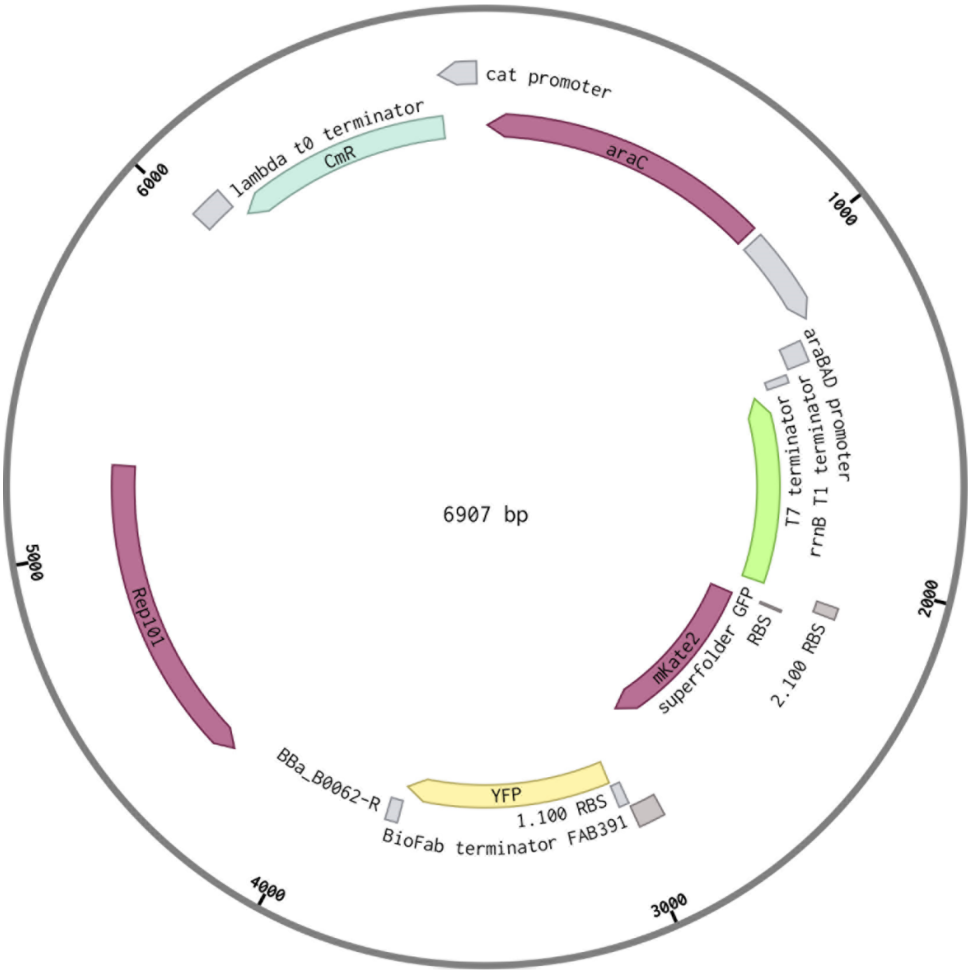

(2) Plasmid map of pBbS8C-mKate2-ccdB. Diverse promoters were cloned in front of the pBbS8C-mKate2 plasmid.

Supplementary Notes 3. Detailed description on the constructing strains with four genes.

Plasmid with four genes were obtained by Golden Gate. To improve DNA assembly efficiency of Golden Gate, five 4-bp linkers (ATCT, GCTG, CGCT, TCAT, and GAGT) with higher affinity and specificity were chosen for Golden Gate because these linkers could significantly enlarge number and accuracy of correct clones [7]. Next, we also equipped the backbone plasmid with mKate2 and ccdB proteins. The terminal of the ccdB and mKate2 cassette was also equipped with ATCT and GAGT, respectively. It is noting that ccdB protein can lock up DNA gyrase by damaging double-stranded DNA, which ultimately triggers the death of negative DH5α with the backbone plasmid [8]. In contrast, the DB3.1 strain with the ccdB resistance gene (gyrA462) was used for backbone perseverance.

Four gene were cloned into the target plasmid as the following process. For instance, genes, including 26E7, 11C1, 9H9 and CHI, were cloned into pCDF plasmids, respectively. These four gene cassettes, including P_{T7} promoter, gene and terminator, were individually equipped with two different linkers. In detail, the 26E7 cassettes was equipped with linkers of ATCT and GCTG. The 11C1 cassettes was equipped with linkers of GCTG and CGCT. The 9H9 cassettes was equipped with linkers of CGCT and TCAT. The CHI cassettes were equipped with linkers of TCAT, and GAGT. Finally, four gene expression elements were inserted into the backbone plasmid by Golden gate.

Promoter library was established as the following procedure. POT-mRFP1 was chosen from lab stock and the detail sequence information was described as previously [11]. This POT-mRFP1 plasmid was used as the original plasmid to construct the candidate forty-eight plasmids, which comprised the diverse combinations of four different genes (26E7, 11C1, 9H9 and CHI) and twelve candidate promoters (Figure). Strain with POT-mRFP1can display red on LB media, whereas the strain with the target plasmid exhibited no color. Thus, diverse strains with forty-eight plasmids presented no color and individually chosen for sanger sequencing. It is noting that gene cassettes of these forty-eight plasmids were equipped with two 4-bp linkers as above described. For instance, ATCT and GCTG were used to replace the original linkers of POT-mRFP1, and chosen for the 26E7 cassettes with diverse promoters.

Supplementary Notes 4. Detailed description on the constructing diverse Flavonoid strains.

We further demonstrated the universal applicability of the engineered naringenin pathway through four cases, including resveratrol, genistein, sakuranetin, and hesperetin.

(1) Resveratrol strain construction and chemical detection.

VvSTS gene was synthesized by GENEWIZ (Suzhou, China) and cloned into pETduet-1 plasmid, resulting in pET-VvSTS. Then this pET-VvSTS plasmid was transformed the competent cells with 26E7 and 11C1, resulting in RES strain.

The process for resveratrol biosynthesis was employed as the following process. Pick out the strain into LB medium and incubate overnight at 30℃. Then 10 μL overnight culture was transferred into the MOPS medium. One millimole isopropyl β-D-1-thiogalactopyranoside (IPTG) was added to the MOPS medium for inducing VvSTS gene expression after the OD_{600} was reached to 0.6. Then incubate the strain at 30℃ for two days.

Detection method of resveratrol was similar to naringenin. In detail, Equivalent ethanol was added to the 300 μL fermentation broth of resveratrol. Fluctuate the mixture for 2 minutes twice and place it at room temperature for 1 hour. Centrifuge the samples at 13000 rpm and 4℃ for 10 minutes. Then the supernatants were analyzed by Agilent 1260 HPLC system. The C18 column (3×100mm 2.7u) was utilized to determine resveratrol by a gradient elution at 30℃ and 0.3 mL/min flow rate: 10% to 40% acetonitrile (vol/vol) for 5 min, 40% acetonitrile (vol/vol) for 7 min, 40% to 95% acetonitrile (vol/vol) for 3 min, 95% to 10% acetonitrile (vol/vol) for 3 min. The detection wavelength of resveratrol was 310 nm.

(2) Genistein strain construction and chemical detection.

2-hydroxyisoflavanone synthase (IFS), cytochrome P450 enzyme reductase (CPR) and 2-hydroxyisoflavanone dehydratase (HID) were required to biosynthesize genistein from naringenin[12]. *Liu et al.* have optimized the different elements of these three genes[12]. Therefore, we constructed the similar plasmids, including pET-KKK-LjtIFS-GmHID and pACYC-OmpAL-LjtCPR.

OmpAL-LjtCPR was synthesized by GENEWIZ (Suzhou, China) and cloned into pACYC-duet plasmid, resulting in pACYC-OmpAL-LjtCPR. KKK-LjtIFS was synthesized by GENEWIZ (Suzhou, China) and cloned into pETduet-1 plasmid, resulting in pET-KKK-LjtIFS. Then, GmHID was inserted into pET-KKK-LjtIFS by Gibson assembly. To complete the genistein biosynthesis pathway, pET-KKK-LjtIFS-GmHID and pACYC-OmpAL-LjtCPR were transformed into the NAR2.0 competent cell, which produced the highest naringenin production in this work. The resulting strain was named as GEN strain.

The process for genistein biosynthesis was employed as the similar process of resveratrol biosynthesis. Detection method of genistein was described as the following procedure. In detail, Equivalent ethanol was added to the 300 μL fermentation broth of resveratrol. Fluctuate the mixture for 2 minutes twice and place it at room temperature for 1 hour. Centrifuge the samples at 13000 rpm and 4℃ for 10 minutes. Then the supernatants were analyzed by Agilent 1260 HPLC system. The C18 column (3×100mm 2.7u) was utilized to determine resveratrol by a gradient elution at 30℃ and 0.3 mL/min flow rate: 10% to 50% methanol (vol/vol) for 6 min, 50% methanol(vol/vol) for 7 min, 50% to 95% methanol (vol/vol) for 3 min, 95% to 10% methanol (vol/vol) for 3 min. The detection wavelength of genistein was 250 nm.

(3) Sakuranetin strain construction and chemical detection.

Other groups have evaluated the candidate O-methyltransferases to biosynthesize sakuranetin from naringenin by methylation process [13]. Two O-methyltransferases, including NOMT[13a] and PfOMT3[13b], were synthesized by GENEWIZ (Suzhou, China) and cloned into pETduet-1 plasmid, resulting in pET-NOMT and pET-PfOMT3. To complete the sakuranetin biosynthesis pathway, pET-NOMT and pET-PfOMT3 were transformed into the NAR2.0 competent cell, respectively. The strains were named as SAK-N and SAK-P.

The process for sakuranetin biosynthesis was employed as the similar process of resveratrol biosynthesis. To increase sakuranetin biosynthesis pathway, 1 g/L methionine, 90 μM pyridoxal 5′ -phosphate (PLP) and 6 mM Mg^{2+} were added in MOPS media as described by *Sun et al* [13b].

Detection method of sakuranetin was similar to that of naringenin procedure. The detection wavelength of sakuranetin was 290 nm.

(3) Hesperetin strain construction and chemical detection.

Flavonoid 3′-hydroxylase (F3′H) from *Gentiana triflora*, cytochrome P450 reductase (CPR) from *Arabidopsis thaliana*, and O-methyltransferase MpOMT from *Mentha piperita* were required to biosynthesize hesperetin from naringenin[14]. *Liu et al.* have optimized the different elements for hesperetin biosynthesis. Therefore, we cloned the similar elements into pET-duet 1 plasmid, resulting in pET-Gt-trF3'H-ATR2-SumoMpOMT-metK. To complete the hesperetin biosynthesis pathway, the resulting plasmid was transformed into NAR2.0 competent cell, which was named as HEP.

The process for hesperetin biosynthesis was employed as the similar process for sakuranetin production. Detection method of hesperetin was similar to that of naringenin procedure.

Supplementary Figures

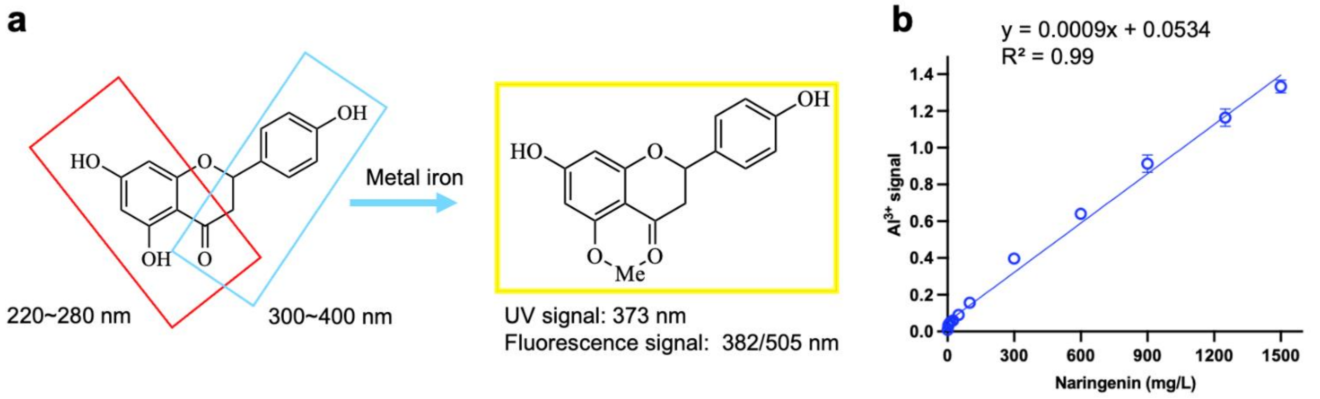

Figure S1. (a) The principle for screening the candidate mutants with higher naringenin production. Naringenin presents specific signals based on the relevant structures. The structure marked by the red box displays the absorption peak from 220 to 280 nm. The structure marked by the blue box displays the absorption peak from 300 to 400 nm. In contrast, the complex by naringenin and metal ions, such as aluminum ions, can present the specific UV absorption peak at 373 nm. This chemical method of naringenin detection has been described previously[1a]. (b) Al^{3+} signals respond to diverse Naringenin concentration.

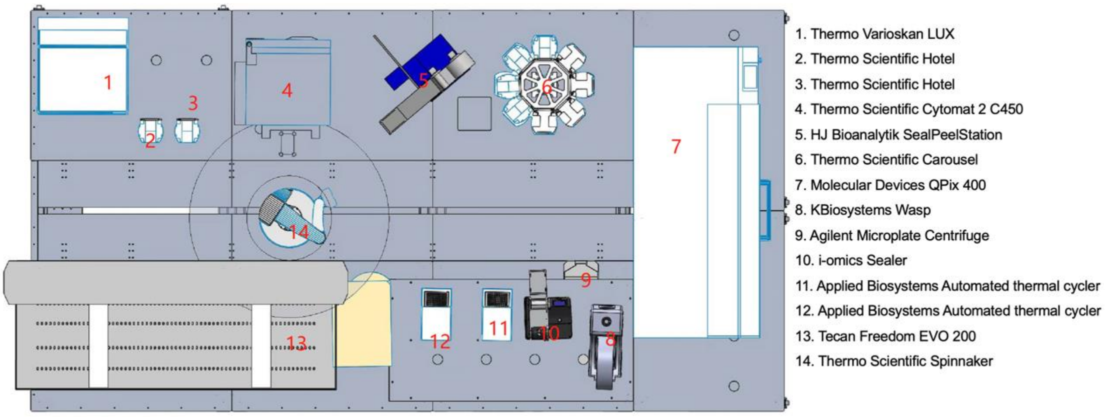

Figure S2. overview of automation workstation in this study. The diverse devices in this automatic workstation were described as follows: (1) Thermo Varioskan LUX (2) Thermo Scientific Hotel (3) Thermo Scientific Hotel (4) Thermo Scientific Cytomat 2 C450 (5) HJ Bioanalytik SealPeelStation (6) Thermo Scientific Carousel (7) Molecular Devices QPix 400 (8) KBiosystems Wasp (9) Agilent Microplate Centrifuge (10) i-omics Sealer (11) Applied Biosystems Automated thermal cycler (12) Applied Biosystems Automated thermal cycler (13) Tecan Freedom EVO 200 (14) Thermo Scientific Spinnaker

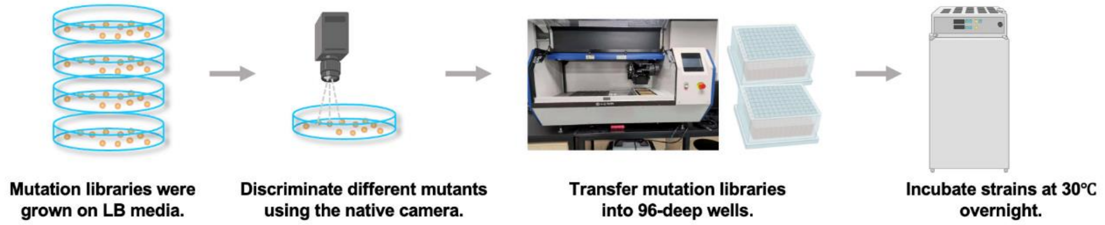

Figure S3. Pick out the candidate mutants by automatic platform. Detail steps have been described in supplementary video 1.

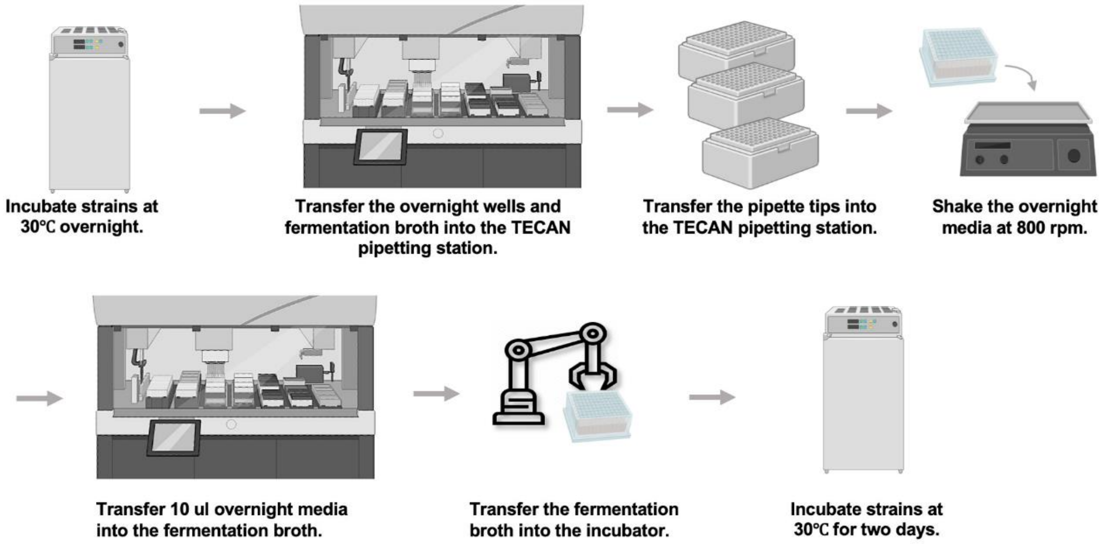

Figure S4. Transfer the overnight media into the new fermentation broth by automatic platform. Detail steps have been described in supplementary video 2.

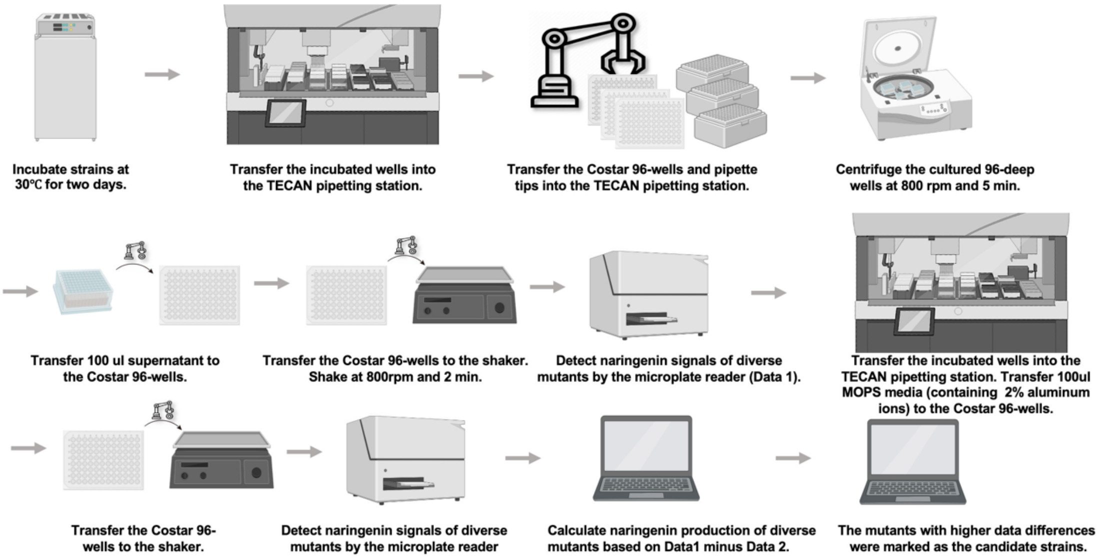

Figure S5. Screen the candidate mutants by automatic platform. Detail steps have been described in supplementary video 3.

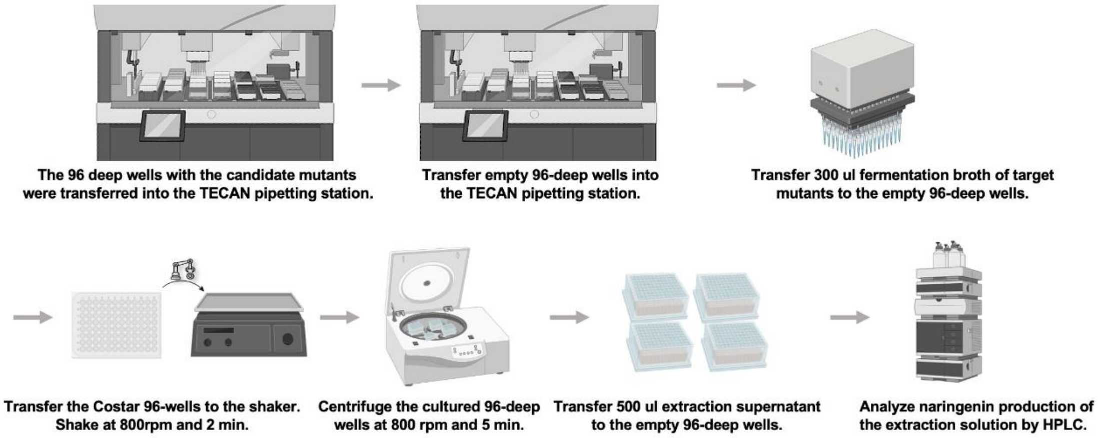

Figure S6. Extract the naringenin product of the candidate mutants by automatic platform. Detail steps have been described in supplementary video 4.

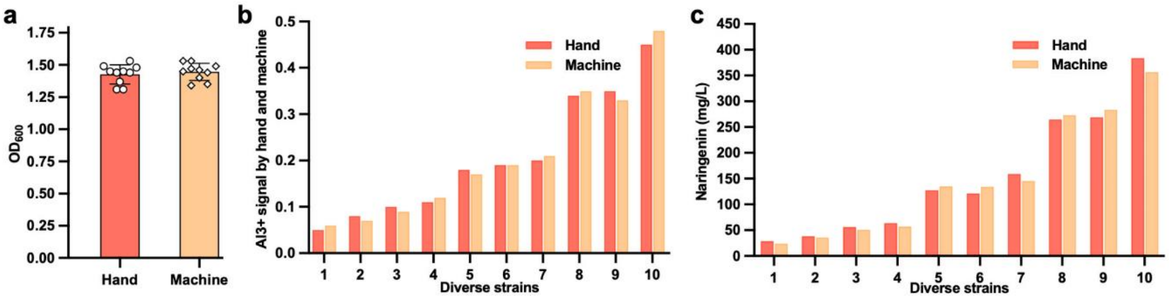

Figure S7. Comparison of different parameters between automation workflow and manual manipulation. (a) Cell density differences between manual and automated colony picking and transfers. Ten strains with diverse naringenin biosynthesis abilities were separately picked out into the wells of 96-deep well plates by hand and using the QPix. The optical density was evaluated after overnight incubation at 30°C and 800 rpm. (b) Analysis of narigenin titers using the Al^{3+} assay for colonies picked manually and using the QPix. Ten strains with diverse naringenin biosynthesis abilities were chosen to evaluate differences between manual and automated screening using the Al^{3+} method. (c) Compound extraction differences between manual and automated methods. Naringenin samples of the above 10 strains were separately extracted manually and using automation after 2 days of incubation in 0.8 mL MOPS media. Note: hand means the corresponding assays were employed by manual manipulation; machine means the corresponding assays were employed by automatic workflow.

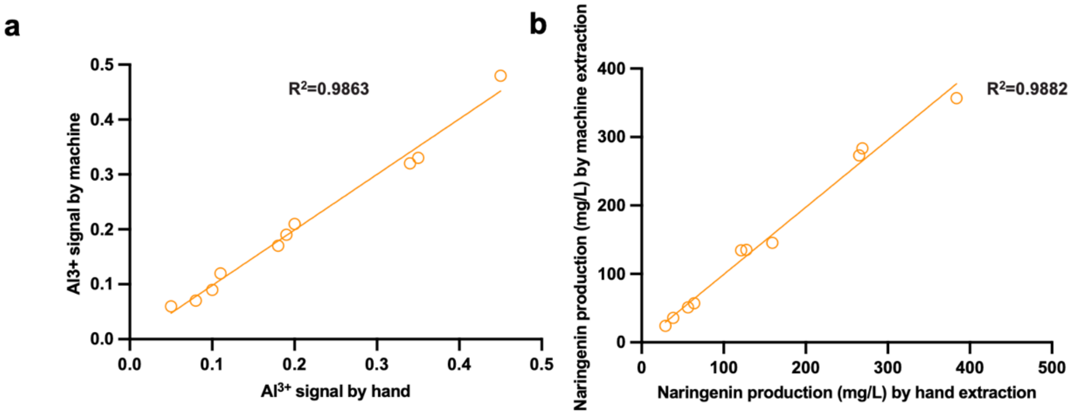

Figure S8. Differences of chemical method screen and naringenin extraction between hand and automation.
(a) Chemical method screen differences between hand and automation. Strains from Figure 1 present diverse naringenin productions. Therefore, ten strains with diverse naringenin biosynthesis abilities were chosen to evaluate chemical method screen differences by hand and automation.
(b) Compound extraction differences between hand and automation.
Naringenin samples of the above ten strains were separately extracted by hand and automation after 2-day incubation in 0.8 mL MOPS media.
Note: hand means the corresponding assays were employed by manual manipulation; machine means the corresponding assays were employed by automatic workflow.

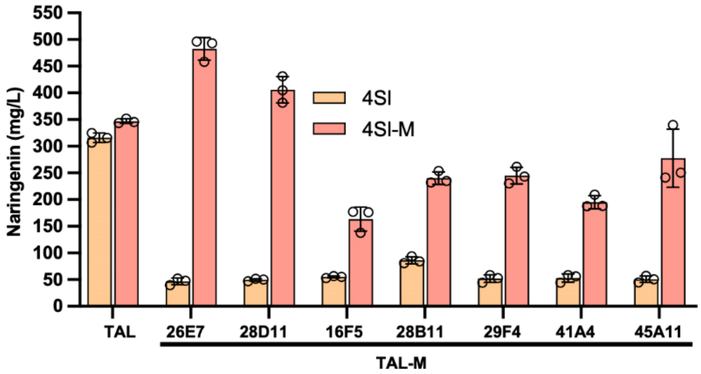

Figure S9. Investigating the potential epistasis of the TAL gene. The TAL gene and its mutants in pBbE5K (ColE1 replicon, 20~30 copies) plasmid were transformed into competent cells with plasmid pCDF-4CL-CHS-CHI (4SI) or pCDF-4CL-11C1-CHS-9H9-CHI (4SI-M), respectively. Naringenin concentrations were determined by HPLC in biological triplicates, and the error bars represent the standard deviation.

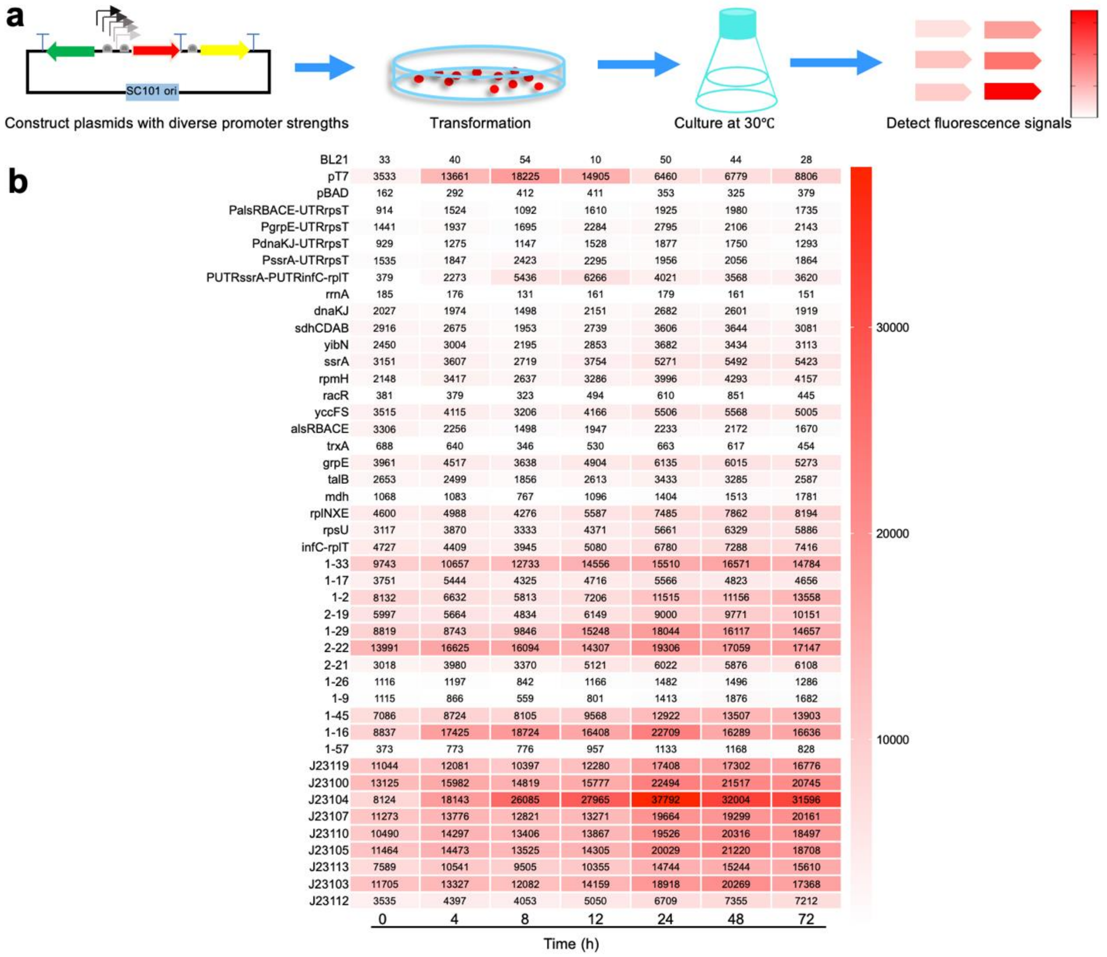

Figure S10. Expression strength levels of the selected promoters in this study.
(a) Workflow for confirming the expression levels of the candidate promoters.
(b) Expression levels of the selected promoters in this study. The P_{T7} and P_{BAD} promoters were used as the positive and negative promoters, respectively.

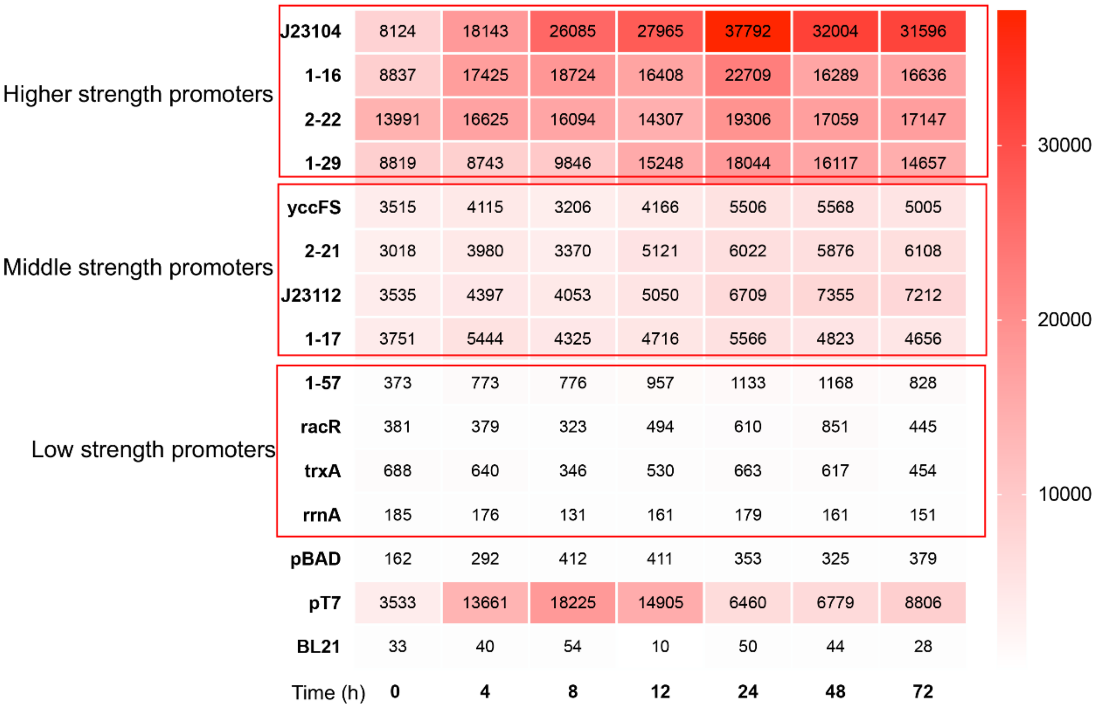

Figure S11. Expression strength levels of the 12 candidate promoters, which were used to activate TAL-26E7, 4CL-11C1, CHS-9H9, and CHI genes. The P_{T7} and P_{BAD} promoters were used as the positive and negative promoters, respectively.

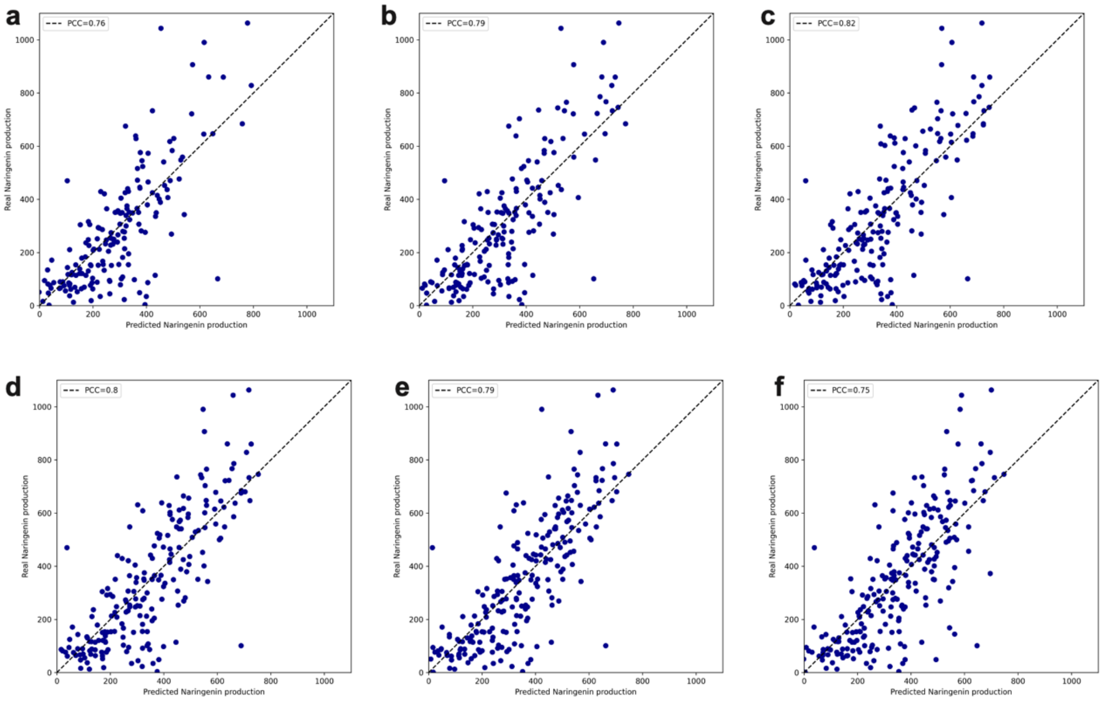

Figure S12. ProEnsemble performance of different enhanced datasets.
(a) Pearson correlation coefficient (PCC) of predicted naringenin production and real production based on the initial dataset and enhanced dataset (with naringenin production>800mg/L).
(b) PCC of predicted naringenin production and real production based on the initial dataset and enhanced dataset (with naringenin production>700mg/L).
(c) PCC of predicted naringenin production and real production based on the initial dataset and enhanced dataset (with naringenin production>600mg/L).
(d) PCC of predicted naringenin production and real production based on the initial dataset and enhanced dataset (with naringenin production>500mg/L).
(e) PCC of predicted naringenin production and real production based on the initial dataset and enhanced dataset (with naringenin production>400mg/L).
(f) PCC of predicted naringenin production and real production based on the initial dataset and enhanced dataset (with all samples).

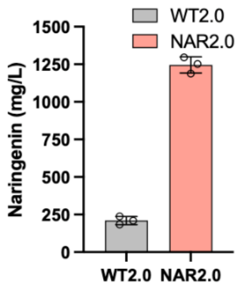

Figure S13. Naringenin production from WT2.0 and NAR2.0. WT2.0 strain with genotype $P_{2\text{-}22}$-TAL-$P_{1\text{-}16}$-4CL-$P_{1\text{-}17}$-CHS-P_{trxA}-CHI; NAR2.0 strain with genotype $P_{2\text{-}22}$-TAL-26E7-$P_{1\text{-}16}$-4CL-11C1-$P_{1\text{-}17}$-CHS-9H9-P_{trxA}-CHI.

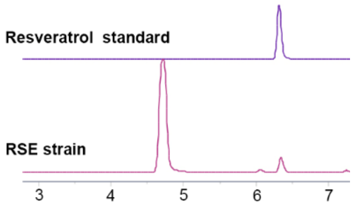

Figure S14. LC chromatograms by resveratrol standard and resveratrol production from RSE strain. Resveratrol samples from the RSE strain were extracted after 48h induction time.

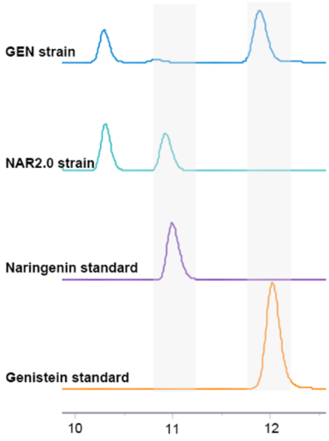

Figure S15. LC chromatograms of genistein and naringenin samples. NAR2.0 strain, which is the highest naringenin strain in this study. GEN strain was obtained by transforming pET-KKK-LjtIFS-GmHID and pACYC-OmpAL-LjtCPR into NAR2.0 competent cells.

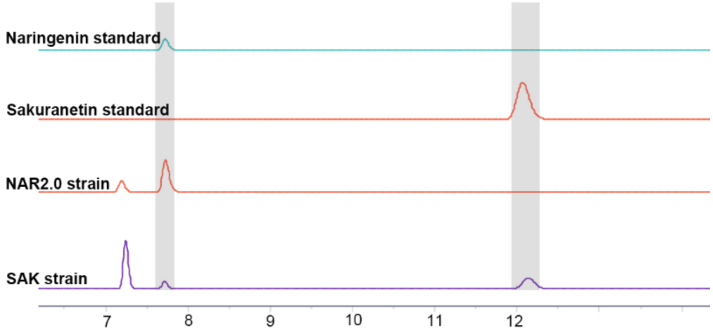

Figure S16. LC chromatograms of sakuranetin and naringenin samples. NAR2.0 strain, which is the highest naringenin strain in this study. SAK strain was obtained by transforming pET-NOMT plamid into NAR2.0 competent cells.

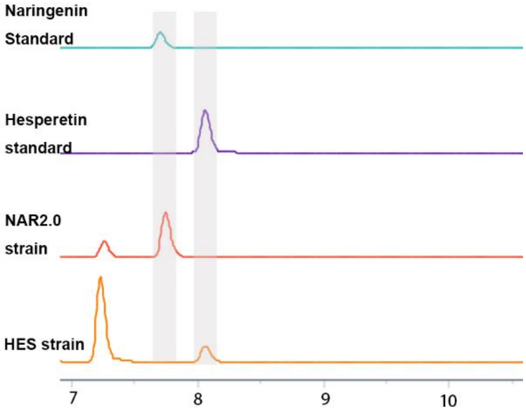

Figure S17. LC chromatograms of Hesperetin and naringenin samples. NAR2.0 strain, which is the highest naringenin strain. HES strain was obtained by transforming pET-Gt-trF3'H-ATR2-SumoMpOMT-metK plasmid into NAR2.0 competent cells.

Supplementary Tables

Table S1. Strains used in this study.

**Strains**	**Relevant information**	**Sources**
DH5*α*	F-ϕ80dlacZ△M15 △(*lacZYAIargF*)*U169deoRrecA1endA1hsdR17*(r_K^- m_K^+) *phoA*△*supE44thi-1gyrA96 relA1*	New England Biolabs
BL21(DE3)	F^- *ompThsdS*$_B$ (rB^-mB^-) *galdcm* (DE3)	Invitrogen
T4SI	BL21(DE3) with plasmid pCDF_TAL_4CL_CHS_CHI	This study
T4S_S8cK	BL21(DE3) with plasmids pCDF-T4S and pBbS8c_RFP	This study
T4S_S8c_TAL	BL21(DE3) with plasmids pCDF-T4S and pBbS8c_TAL	This study
T4S_a8cK	BL21(DE3) with plasmids pCDF-T4S and pbba8c_RFP	This study
T4S_a8c_TAL	BL21(DE3) with plasmids pCDF-T4S and pbba8c_TAL	This study
T4S_b8aK	BL21(DE3) with plasmids pCDF-T4S and pbbb8a_GFP	This study
T4S_b8a_TAL	BL21(DE3) with plasmids pCDF-T4S and pbbb8a_TAL	This study
T4S-E5KK	BL21(DE3) with plasmids pCDF-T4S and pBbE5K_RFP	This study
T4S-E5K_TAL	BL21(DE3) with plasmids pCDF-T4S and pBbE5K_pBAD-TAL	This study
T4S-RSFK	BL21(DE3) with plasmids pCDF-T4S and pRSF_Duet-1	This study
T4S-RSF_TAL	BL21(DE3) with plasmids pCDF-T4S and pRSF_pBAD-TAL	This study
TSI_S8cK	BL21(DE3) with plasmids pCDF-TSI and pBbS8c_RFP	This study
TSI_S8c_4CL	BL21(DE3) with plasmids pCDF-TSI and pBbS8c_4CL	This study
TSI_a8cK	BL21(DE3) with plasmids pCDF-TSI and pbba8c_RFP	This study

TSI_a8c_4CL	BL21(DE3) with plasmids pCDF-TSI and pbba8c_4CL	This study
TSI_b8aK	BL21(DE3) with plasmids pCDF-TSI and pbbb8a_GFP	This study
TSI_b8a_4CL	BL21(DE3) with plasmids pCDF-TSI and pbbb8a_4CL	This study
TSI-E5KK	BL21(DE3) with plasmids pCDF-TSI and pBbE5K_RFP	This study
TSI-E5K_4CL	BL21(DE3) with plasmids pCDF-TSI and pBbE5K_pBAD-4CL	This study
TSI-RSFK	BL21(DE3) with plasmids pCDF-TSI and pRSF_Duet-1	This study
TSI-RSF_4CL	BL21(DE3) with plasmids pCDF-TSI and pRSF_pBAD-4CL	This study
T4I_S8cK	BL21(DE3) with plasmids pCDF-T4I and pBbS8c_RFP	This study
T4I_S8c_CHS	BL21(DE3) with plasmids pCDF-T4I and pBbS8c_CHS	This study
T4I_a8cK	BL21(DE3) with plasmids pCDF-T4I and pbba8c_RFP	This study
T4I_a8c_CHS	BL21(DE3) with plasmids pCDF-T4I and pbba8c_CHS	This study
T4I_b8aK	BL21(DE3) with plasmids pCDF-T4I and pbbb8a_GFP	This study
T4I_b8a_CHS	BL21(DE3) with plasmids pCDF-T4I and pbbb8a_CHS	This study
T4I-E5KK	BL21(DE3) with plasmids pCDF-T4I and pBbE5K_RFP	This study
T4I-E5K_CHS	BL21(DE3) with plasmids pCDF-T4I and pBbE5K_pBAD-CHS	This study
T4I-RSFK	BL21(DE3) with plasmids pCDF-T4I and pRSF_Duet-1	This study
T4I-RSF_CHS	BL21(DE3) with plasmids pCDF-T4I and pRSF_pBAD-CHS	This study
T4S_S8cK	BL21(DE3) with plasmids pCDF-T4I and pBbS8c_RFP	This study
T4S_S8c_CHI	BL21(DE3) with plasmids pCDF-T4I and pBbS8c_CHI	This study
T4S_a8cK	BL21(DE3) with plasmid pCDF-T4I and pbba8c_RFP	This study
T4S_a8c_CHI	BL21(DE3) with plasmids pCDF-T4I and pbba8c_CHI	This study
T4S_b8aK	BL21(DE3) with plasmids pCDF-T4I and pbbb8a_GFP	This study
T4S_b8a_CHI	BL21(DE3) with plasmids pCDF-T4I and pbbb8a_CHI	This study
T4S-E5KK	BL21(DE3) with plasmids pCDF-T4I and pBbE5K_RFP	This study
T4S-E5K_CHI	BL21(DE3) with plasmids pCDF-T4I and pBbE5K_pBAD-CHI	This study
T4S-RSFK	BL21(DE3) with plasmids pCDF-T4I and pRSF_Duet-1	This study
T4S-RSF_CHI	BL21(DE3) with plasmids pCDF-T4I and pRSF_pBAD-CHI	This study
NAR1.0	The strain with the highest naringenin production from the first collecting dataset, which was used for machine learning	This study
NAR2.0	The strain with the highest naringenin production from machine learning	This study
RES	pET-VvSTS was transformed into BL21(DE3) with 26E7 and 11C1 genes, which was used for resveratrol biosynthesis.	This study
GEN	Strain #2-1 with plasmids pET-KKK-LjtIFS-GmHID and pACYC-OmpAL-LjtCPR was used for genistein biosynthesis.	This study
SAK-N	Strain #2-1 with plasmid pET-NOMT was used for sakuranetin biosynthesis.	This study

SAK-P	#2-1 with plasmid pET-PfOMT3 was used for sakuranetin biosynthesis.	This study

Table S2. Plasmids used in this study.

Plasmids	Relevant information	Notes
pBbS8c_RFP	Cm^R, SC101 origin of replication	Lab stock
pBbS8C-TAL	TAL was cloned into pBbS8C plasmid	This study
pBbS8C-4CL	4CL was cloned into pBbS8C plasmid	This study
pBbS8C-CHS	CHS was cloned into pBbS8C plasmid	This study
pBbS8C-CHI	CHI was cloned into pBbS8C plasmid	This study
pBba8C_RFP	Cm^R, p15a origin of replication	Lab stock
pBba8C-TAL	TAL was cloned into pBba8C plasmid	This study
pBba8C-4CL	4CL was cloned into pBba8C plasmid	This study
pBba8C-CHS	CHS was cloned into pBba8C plasmid	This study
pBba8C-CHI	CHI was cloned into pBba8C plasmid	This study
pBbb8a_RFP	Amp^R, BBR1 1 origin of replication	Lab stock
pBbb8a-TAL	TAL was cloned into pBbb8a plasmid	This study
pBbb8a-4CL	4CL was cloned into pBbb8a plasmid	This study
pBbb8a-CHS	CHS was cloned into pBbb8a plasmid	This study
pBbb8a-CHI	CHI was cloned into pBbb8a plasmid	This study
pBbE5K	$Kana^R$, colE1 origin of replication	Lab stock
pBbE5K-pBAD-TAL*	The cassette containing pBAD promoter and TAL gene were cloned into pBbE5K plasmid.	This study
pBbE5K-pBAD-4CL	The cassette containing pBAD promoter and 4CL gene were cloned into pBbE5K plasmid	This study
pBbE5K-pBAD-CHS	The cassette containing pBAD promoter and CHS gene were cloned into pBbE5K plasmid	This study
pBbE5K-pBAD-CHI	The cassette containing pBAD promoter and CHI gene were cloned into pBbE5K plasmid.	This study
pRSFDuet-1	$Kana^R$, RSF origin of replication	Novagen
RSF-pBAD-TAL*	The cassette containing pBAD promoter and TAL gene were cloned into RSF plasmid	This study
RSF-pBAD-4CL	The cassette containing pBAD promoter and 4CL gene were cloned into RSF plasmid	This study
RSF-pBAD-CHS	The cassette containing pBAD promoter and CHS gene were cloned into RSF plasmid	This study
RSF-pBAD-CHI	The cassette containing pBAD promoter and CHI gene were cloned into RSF plasmid	This study
pCDFDuet-1	Str^R, CloDF13 origin of replication	Novagen
pCDF-T4SI	TAL, 4CL, CHS and CHI were cloned into the CDF plasmid	This study
pCDF-T4I	TAL, 4CL and CHI were cloned into the CDF plasmid	This study
pCDF-TSI	TAL, CHS and CHI were cloned into the CDF plasmid	This study

pCDF-4SI	4CL, CHS and CHI were cloned into the CDF plasmid	This study
pETduet-1	Amp^R, ColE1 origin of replication	Novagen
pET-VvSTS	VvSTS was cloned into pETduet-1 plasmid	This study
pET-KKK-LjtIFS-GmHID	KKK-LjtIFS and GmHID were cloned into pETduet-1 plasmid	This study
pACYCduet-1	Cm^R, p15a origin of replication	Novagen
pACYC-OmpAL-LjtCPR	OmpAL-LjtCPR was cloned into pACYCduet-1 plasmid.	This study
pET-NOMT	NOMT was cloned into pETduet-1 plasmid	This study
pET-PfOMT3	PfOMT3was cloned into pETduet-1 plasmid	This study

Note:
*: the plasmids, such as pBbE5K-pBAD-TAL and RSF-pBAD-TAL were constructed as described by Supplementary Notes 1.

Table S3. Mutation sites and naringenin production in the candidate mutants.

Number	Al^{3+} signal	NAR (mg/L) a*	NAR (mg/L) b*	Mutation information
TAL-K	0.15	55.13	54.67±1.64	ND
16F5	0.52	396.52	325.10±11.87	CAA1224(**Gln**)>CAT(**His**); ATC2037(Ile)>ATA(Ile)
26E7	0.45	307.86	373.61±28.25	CAC522(**His**)>CAA(**Gln**); 583CTG(Leu)>TTG(Leu)<br>GCG789(Ala)>GCC(Ala); CAC879(His)>CAT(His)
28D11	0.41	294.52	340.90±18.80	AG26C(**Ser**)>ATC(**Ile**)
29F4	0.35	249.08	230.34±20.02	CTG132(Leu)>CTA(Leu);1903CTG(**Leu**)>ATG(**Met**)
40B11	0.30	233.19	237.65±9.08	AG800C(**Ser**)>ATC(**Ile**)
41A4	0.27	204.12	192.97±19.92	CCG1288(**Pro**)>TCG(**Ser**); GTT1652(**Val**)>GAT(**Asp**)
45A11	0.25	198.94	215.20±15.21	AA1667G(**Lys**)>ACG (**Thr**)
4CL-K	0.08	44.89	45.41±2.99	ND
11C1	0.21	134.57	134.82±17.11	CT196G(**Leu**)>CCG (**Pro**); GTT505(Val)>GTC(Val)
2C1	0.20	111.47	126.05±26.71	CTG32 (Leu)>CTT(Leu); CT196G(**Leu**)>CCG (**Pro**)
24H10	0.18	68.42	76.20±8.01	GG119C(**Gly**)>GTC(**Val**)
35D5	0.16	67.19	63.46±7.92	586CTG588(**Leu**)>GTT(**Val**)
45C7	0.19	70.03	58.76±10.88	GCA468(Ala)>GCG(Ala); G616GT(**Gly**)>AGT(**Ser**);<br>GTG1437(Val)>GTA(Val)
CHS-K	0.05	28.98	32.43±3.72	ND
9H9	0.21	149.27	156.50±16.28	877TCC(**Ser**)>ACC(**Thr**); CCG963(Pro)>CCT(Pro)
23F6	0.15	66.02	63.82±8.84	AA437A(**Lys**)>AGA(**Arg**); GA837A(**Glu**)>GTA(**Val**)

Notes: automatic biofoundry screen out the candidate mutants by the increased Al^{3+} signal than that of the wild-genotype strain; ND means no mutation site when the nucleotides of the wild-type gene were sequenced.
a*: naringenin production and mutation information of the candidate mutants were determined by HPLC method and sanger sequencing.
b*: the coding sequences of candidate mutants were reconstructed into the control plasmid, pBbS8c_RFP and transformed into the corresponding competent cells (described as Supplementary Table 2). Naringenin productions of these mutants were further confirm by HPLC method.

Table S4. Kinetic properties of TAL and the mutants.

Genes a*	K_m (mM)	K_{cat} (s^{-1})	K_{cat}/K_m($mM^{-1}\cdot s^{-1}$)	References
RgTAL	0.38	114.00	300.00	[2]
16F5(Q408H)	1.63	1268.00	776.48	This study
28D11(S9I)	2.16	2372.00	1097.13	This study
26E7(H174Q)	2.09	2416.00	1158.20	This study
RgTAL (A11T)	0.38	142	375	[2]
RgTAL (E518V)	0.29	110	385	[2]

RgTAL (S9N/A11T/E518V)	0.29	111	391	[2]
SbPAL1	0.12	0.31	2.52	[15]
SbPAL1 (F102Y)	2.89	1.11	0.38	[15]
ZmPAL	41	0.94	0.02	[16]
AtTAL	1.05	0.08	0.08	[17]
AtTAL (F144H)	0.53	0.75	1.42	[17]
RbaTAL	15.60	27.70	1.77	[18]
RbaTAL (H89F)	1.54	87.00	56.49	[18]

Note: naringenin production of RgTAL-16F5, RgTAL-28D11, and RgTAL-26E7 is closely to that of the positive control strain with plasmids pBbE5K-TAL and pCDF-4CL-CHS-CHI (Fig. 3). Therefore, we evaluated the kinetic properties of these enzymes. The highest naringenin production and the optimal K_{cat}/K_m value were determined for RgTAL-26E7 among these three mutants. Therefore, RgTAL-26E7 was chosen for the following experiments.
RgTAL (KF765779) from *Rhodotorula glutinis*; SbPAL1 from *Sorghum bicolor*; ZmPAL from *Zea mays L.*; AtTAL from *Arabidopsis thaliana*; RbaTAL from *Rhodobacter capsulatus*.

Table S5. Kinetic properties of 4CL and the mutants

Genes	K_m (mM)	K_{cat} (s^{-1})	K_{cat}/K_m($mM^{-1}\cdot s^{-1}$)	References
4CL	0.65	$3.01*10^6$	$4.63*10^3$	This study
11C1 (L66P)	0.06	$5.75*10^6$	$9.58*10^3$	This study
Pn4CL1	23.33	0.70	30.00	[19]
Pn4CL3	13.70	0.81	59.12	[19]
Nt4CL2	1.50	3.98	$2.65*10^3$	[20]
To4CL	7.47	0.66	0.09	[3]
Sm4CL1	11.89	0.07	5.89	[21]
Sm4CL2	19.67	0.71	36.10	[21]
Pl4CL1	3.51	1.02	$0.29*10^3$	[22]
Pl4CL2	9.10	1.28	$0.14*10^3$	[22]

Pn4CL3 and Pn4CL1 from *Piper nigrum*; Nt4CL2 from *Nicotiana tabacum*; To4CL from tomato; Sm4CL1 and Sm4CL2 from *Selaginella moellendorffii*.

Table S6. Kinetic properties of CHS and the mutants.

Genes	K_m (mM)	K_{cat} (s^{-1})	K_{cat}/K_m($mM^{-1}\cdot s^{-1}$)	References
CHS	0.42	143.60	344.78	This study
9H9 (S293T)	0.59	853.20	1435.16	This study
SmCHS	2.58	0.13	0.05	[23]
GbCHS	31.10	0.74	0.02	[23]
OsCHS-1	76.30	0.32	0.00	[23]
AmCHS	49.70	1.27	0.03	[23]
MsCHS	6.10	0.09	0.01	[24]
PcCHS	13.6	0.11	0.01	[25]

SmCHS from *Selaginella moellendorffii;* GbCHS from *Ginkgo biloba*; OsCHS-1 from rice; MsCHS from *Medicago sativa* (alfalfa); PcCHS from *Polygonum cuspidatum*.

Table S7. Encode the 12 candidate promoters for machine learning.

Code number	Promoter
0	23104
1	1-16
2	2-22
3	1-29
4	yccFS
5	2-21
6	23112
7	1-17
8	1-57
9	racR
10	trxA
11	rrnA

Note: the 12 candidate promoters were encoded from 0 to 11 based on their expression strength levels.

Table S8. Sequences of E. coli-codon optimized synthetic genes.

Genes	Condon optimized sequences
TAL	atggcgccgcgcccgacttctcaaagccaggcccgcacttgcccgaccacccaggttacccaagttgatatcgttgagaaaatgctggcggctccgactgatagcaccctggagctggacggttatagcctg<br>aacctgggtgatgttgtgagcgctgcgcgtaagggtcgtccggttcgtgttaaagatagcgatgaaatccgcagcaaaatcgacaagagcgttgaatttctgcgcagccaactgagcatgtctgtttacggtgtg<br>accaccggctttggcggctccgcggacacccgcaccgaggacgcaattagcctgcaaaaggcgctgctggaacaccagctgtgtggtgtgctgccgagcagcttcgacagctttcgcctgggtcgtggtct<br>ggagaacagcctgccgctggaagttgttcgcggtgcaatgaccattcgtgtgaactctctgacccgtggccatagcgctgttcgtctggttgttctggaagcactgaccaactttctgaaccacggtattaccccg<br>attgttccgctgcgcggtaccatctccgcgagcggcgatctgtctccactgtcttacattgcagcggcgattagcggtcacccggatagcaaagttcacgtggttcatgaaggcaaagagaagatcctgtacgc<br>gcgcgaagcgatggcgctgtttaacctggagccggtggttctgggtccgaaggagggcctgggtctggtgaacggtaccgcagtttccgcgagcatggcaaccctggcactgcacgacgcgcacatgctg<br>agcctgctgagccaatctctgaccgcgatgaccgtggaggcgatggttggtcacgcgggcagcttccatccattcctgcacgatgttacccgtccgcacccgacccaaatcgaggttgcgggtaacattcgca<br>aactgctggagggctctcgcttcgcggttcaccacgaggaagaggttaaggttaaggatgatgaaggcattctgcgtcaggatcgttatccgctgcgcaccagcccgcaatggctgggtccgctggtgtccg<br>acctgattcacgctcatgccgttctgaccatcgaagcgggtcaaagcaccaccgataacccactgatcgatgttgagaacaagaccagccatcacggtggcaactttcaagcggcagcggttgccaacactat<br>ggaaaagacccgtctgggcctggcccaaatcggtaaactgaacttcacccagctgaccgagatgctgaacgcgggcatgaaccgtggcctgccgagctgcctggcggctgaagacccatccctgagctat<br>cattgcaaaggtctggacattgcggcggctgcatataccagcgaactgggccacctggctaacccggttaccacccacgttcaaccggctgaaatggcaaaccaggcggtgaacagcctggcgctgattag<br>cgcacgtcgtaccaccgaatctaacgacgttctgtccctgctgctggcaacccacctgtactgcgtgctgcaggcgatcgacctgcgtgcgattgagttcgagttcaagaaacagtttggtccggccattgttag<br>cctgatcgaccaacactttggtagcgcgatgaccggtagcaacctgcgtgatgagctggttgaaaaggttaacaagactctggccaagcgtctggagcaaaccaacagctacgatctggttccgcgctggca<br>cgacgcttttagcttcgctgcaggcactgttgttgaggttctgtccagcaccagcctgagcctggcggccgtgaacgcatggaaggttgcggcagccgagagcgcgatctccctgacccgccaggttcgtga<br>aaccttttggtccgctgcaagcacctccagcccggcgctgtcttacctgagcccgcgcacccagatcctgtacgcatttgtgcgtgaggaactgggtgttaaagcccgccgtggtgacgttttcctgggtaaac<br>aagaagttaccatcggcagcaacgttagcaagatttacgaagccatcaagagcggccgtatcaacaacgttctgctgaagatgctggcataa

4CL	atgggtgactgcgttgccccgaaagaggatctgatcttccgcagcaaactgccggacatttacattccaaagcatctgccgctgcatacctattgttttgagaacatcagcaaggttggcgacaagagctgtctgatcaacggcgcaaccggcgaaacctttacctacagccaggttgagctgctgtcccgtaaagttgccagcggcctgaacaagctgggcattcaacaaggtgataccattatgctgctgctgccgaactccccggagtactttttcgctttcctgggtgcgagctatcgcggtgcaatcagcactatggcgaacccattctttaccagcgcagaagtgatcaagcaactgaaagcgagccaagcgaagctgattatcacccaggcatgctatgttgacaaggttaaggactacgcagcggagaaaaacatccagatcatttgtattgacgatgcaccgcaggattgcctgcactttagcaagctgatggaagcggatgagagcgaaatgccggaagtggttattaacagcgatgatgtggtggcactgccgtacagctctggcaccaccggcctgccgaaaggcgttatgctgacccacaagggtctggttaccagcgttgcacaacaggtggatggtgataacccgaacctgtatatgcactccgaggatgttatgatctgcatcctgccactgttccatatctatagcctgaacgctgttctgtgttgtggtctgcgtgcgggcgttaccattctgatcatgcaaaagttcgacattgtgccgtttctggagctgattcagaagtataaggttaccattggtccgtttgttccgccgatcgtgctggccatcgcgaaaagcccggttgttgacaagtacgacctgtctagcgtgcgcaccgttatgagcggtgcagcgccgctgggtaaagagctggaggacgctgttcgtgcgaaattcccgaacgcgaagctgggtcaaggctatggcatgaccgaagccggtccggttctggcgatgtgtctggcgttcgccaaagagccgtatgagattaagtctggcgcatgcggtaccgttgtgcgtaacgccgagatgaaaatcgttgacccagaaaccaacgcgtctctgccgcgtaaccagcgtggtgagatttgcatccgtggtgatcagattatgaaaggttacctgaacgacccggaaagcacccgcaccaccatcgacgaagagggttggctgcacaccggtgacattggtttcatcgacgatgacgatgaactgttcattgttgatcgtctgaaagaaatcattaagtacaaaggttttcaagttgctccggcggagctggaagcactgctgctgacccacccgaccatcagcgatgccgcggtggttccgatgattgacgagaaagcgggtgaagtgccagtggcgtttgttgtgcgtaccaacggttttaccaccaccgaagaagaaatcaaacaatttgtgagcaaacaggttgtgttctacaaacgtatcttccgcgttttcttcgttgacgctattccgaaatccccgagcggcaagattctgcgtaaggatctgcgcgctcgtattgcgagcggcgacctgccgaagtaa
CHS	atggttacggtggaagaataccgcaaagctcaacgcgctgaaggcccggcgacggtgatggcgattggcacggcaaccccgacgaactgtgttgatcagagcacctatccggactattactttcgtatcaccaactctgaacataaaacggatctgaaagaaaaaattcaaacgtatgtgcgaaaaaagcatgatcaaaaaacgctatatgcacctgaccgaagaaattctgaaagaaaatccgagcatgtgtgaatacatggcaccgtctctggatgctcgccaggacattgtggttgtcgaagtgccgaaactgggtaaagaagcggcccagaaagcgatcaaagaatggggccaaccgaaatcaaaaattacccatctggtcttttgcaccacgtcgggtgtggatatgccgggttgtgactatcaactgacgaaactgctgggtctgcgtccgagcgtgaaacgcctgatgatgtaccagcaaggctgcttcgcaggcggtaccgttctgcgtctggcgaaagatctggccgaaaacaataaaggtgcgcgtgttctggtggtgtgtagtgaaatcaccgctgttacgtttcgtggtccgaacgatacgcacctggactccctggttggccaggccctgttcggtgatggtgcaggtgccattatcattggtagcgacccgattccgggcgttgaacgtccgctgtttgaactggtcagcgcagctcaaaccctgctgccggatagccacggcgcaattgacggtcacctgcgtgaagtcggtctgacgttccatctgctgaaagatgtgccgggcctgatctcaaaaaacattgaaaaaagcctggaagaagcgtttcgcccgctgagtatctccgattggaacagcctgttctggattgcacatccgggcggcccggcaatcctggaccaggtcgaaattaaactgggtctgaaaccggaaaaactgaaagcgacccgtaatgttctgtcaaactacggcaatatgagctctgcctgcgtcctgtttattctggatgaaatgcgcaaagcatcggctaaagaaggtctgggcaccacgggtgaaggcctggaatggggcgtgctgttcggctttggtccgggtctgacggtggaaacggtggttctgcatagtgtggctacctaa
CHI	atggcagcaagcattacggcaatcacggttgaaaatctggaatatccggcggtcgttacctctccggtcacgggcaaatcatactttctgggcggtgccggtgaacgtggtctgaccattgagggtaactttatcaaattcacggcaattggcgtttatctggaagatatcgcggtcgcctcactggcggccaaatggaaaggtaaaagctctgaagaactgctggaaaccctggatttttaccgtgacattatctcaggcccgttcgaaaaactgatccgtggttcgaaaattcgcgaactgagcggcccggaatattctcgcaaagtcatggaaaaactgcgtggctcatctgaaatccgtcggcacgtacggtgacgcagaagctgaagcgatgcagaaatttgccgaagcattcaaaccggtgaattttccgccgggtgccagtgttttctatcgtcaatccccggatggcatcctgggtctgtcattttcgccggacaccagcatcccggaaaaagaagcagctctgattgaaaataaagctgtgagttccgcggttctggaaacgatgattggcgaacacgcggtttctccggatctgaaacgctgtctggctgctcgcctgccggctctgctgaatgaaggtgcctttaaaatcggtaactga
VvSTS	atggcttcagtcgaggaatttagaaacgctcaacgtgccaagggtccggccaccatcctagccattggcacagctacccccgaccactgtgtctaccagtctgattatgctgatttctatttcagggtcactaagagcgagcacatgactgcgttgaagaagaagttcaatcgcatatgtgacaaatccatgatcaagaagcgttacattcatttgaccgaagaaatgcttgaggagcacccaaacattggtgcttatatggctccatctcttaacatacgccaagagattatcactgccgaggtacccaagctcggtaaggaagcagcgttgaaggctcttaaagagtggggtcagcctaaatcgaagatcacccaccttgtattttgtaccacctcaggtgtagaaatgcctggtgcagattataaactcgctaatctcttaggcctcgaaccatctgtcagaagagtgatgttgtaccatcaagggtgctatgcaggtggaactgtccttcgaactgctaaggatcttgcagagaataatgcaggagcacgagttcttgtggtgtgctctgagatcacagttgttacatttcgcggcccttccgaagatgctttggactctttagttggccaagcccttttggtgatgggtctgcagctgtaatcgtaggatcagatccggatatctcaattgaacgaccactcttccagcttgtctcagcagcccaaacatttattcctaattctgcaggtgccattgcaggaaacttacgtgaggtgggactcacctttcatttgtggcccaatgtgcccactttaatttctgagaacatagagaaatgtttgactcaggcttttgacccacttggtattagcgattggaactcgttattttggattgctcacccaggtggccctgcaattcttgatgcagttgaagcaaaactcaatttagataaaaagaaactcgaagcaacgaggcatgtgctaagtgagtatggaaacatgtcaagtgcatgtgtgttgtttattttggatgagatgagaaagaaatcccttaaggggggagagggccaccacgggtgaaggattggattggggagtattattcggttttggaccaggcttgactattgaaactgttgtgttacatagcattcctatggttacaaattaagcc
KKK-LjtIFS	atgaagaagaagaccccgactgccaagagcaaagcgctgcgtcatctgccgaatccgccgtccccgaagccgcgtttgccgttcgttggtcacttgcacctgctggaccagccgctcttgcacaactcgttgatcaaattgggtgagaaatacggcccgctctacaccctgtatttcggtagcatgccgacggttgttgcgagcaccccggaattatttaagctgttcctgcaaacccatgaagcaaccagctttagcacgcgcttccagacctccgcgatccgtcgtttaacgtatgacaactcagtggctatggttccgttcgcaccgtactggaagttcatccgcaaggttattatgaatgatctgctgaacgcgactacagtcaacaaactgcgtccactgcgtagccaggagattagaaaagttcttaaggcgatggcacagtctgcggagagccaaaaaccgttgaacgtgacggaagagttacttaaatggaccaatagcaccatcagccgtatgatgctgggtgaagcagagcacgttaaggacattgttcgtgaagttttgaaaatcttcggcgagtattctctgacggattttatctggccacttaagaaactgagagtcggacaatatgaaaaacgcattgatgaaattttttaacaaatttgatccggtgattgaaaaagtgatcaaaaagcgccaagaaatcattaagcgccgtaaagaacgtaatggcgaactagaggagggtgagcagtctgtagtatttctggataccctgttggagtatgcggcagacgagaacatggaaattaagataaccaaagagcagatcaagggactggtggtggatttcttcagcgctggcaccgacagcaccgcggttgccaccgactgggccttggcggagctgattaacaacccgcgcgttctcaagaaggctcgtgaagaggtggatagcgttgtcggtaaagaccgcctggtggatgaaagcgacatccaaaacctgccgtacatccgcgccatcgtgaaagaaacctttcgtatgcacccgcctctgccggttgtaaaaagaaagtgcgtccaagagtgcgaattgaatggttacgtgatcccggaaggtgcgctggttttgttcaacgtgtgggcagtgcaacgtgacccgaagtactggaaaaccccgctggagttccgtccagagcgctttctggaggaagcggacattgacctgaagggccaacattttgagctgctgccattcggttccggtcgtcgcatgtgtccgggtgtcaacctggcgactgccggcatggcgacgctgctgagctcggtcattcagtgttttgaactgcaagtggtgggccctaaaggccaaattctgaagggttctgatgccaaagctaatatggaggagcgtgctggcctgaccgtgccgcgtgcgaacagcctgatgtgcgttccgttggcgcgttccaaggtggctgcggagctcctgagttcataa
GmHID	atggcgaaagaaattgtgaaagaactgctgccgctgattcgcgtgtataaagatggcagcgtggaacgcctgctgagcagcgaaaacgtggcggcgagcccggaagatccgcagaccggcgtgagcagcaaagatattgtgattgcggataacccgtatgtgagcgcgcgcatttttctgccgaaaagccatcataccaacaacaaactgccgatttttctgtattttcatggcggcgcgttttgcgtggaaagcgcgtttagcttttttgtgcatcgctatctgaacattctggcgagcgaagcgaacattattgcgattagcgtggattttcgcctgctgccgcatcatccgattccggcggcgtatgaagatggctggaccaccctgaaatggattgcgagccatgcgaacaacaccaacaccaccaacccggaaccgtggctgctgaaccatgcggattttaccaaagtgtatgtgggcggcgaaacgagcggcgcgaacattgcgcataacctgttactgcgcgcgggcaacgaaagcctgccgggcgatctgaaaattctgggcggcctgctgtgctgcccgtttttttggggcagcaaaccgattggcagcgaagcggtggaaggccatgaacagagcctggcgatgaaagtgtggaactttgcgtgcccggatgcgccgggcggcattgataacccgtggattaacccgtgcgtgccgggcgcgccgagcctggcgaccctggcgtgcagcaaactgctggtgaccattaccggcaaagatgaatttcgcgatcgcgatattctgtatcatcataccgtggaacagagcggctggcaaggcgaactgcagctgtttgatgcgggcgatgaagaacatgcgtttcagctgtttaaaccggaaacccatctggcgaaagcgatgattaaacgcctggcgagctttctggtgtaa
OmpAL-LjtCPR	atgaaaaagacagctatcgcgattgcagtggcactggctggtttcgctaccgtagcgcaggccgaggaaagcagcagcatgaaaatcagcccgctggatctgatgagcgcgatgattaagggcaccctggacccgagcaacgtgagcagcaccagcggtgcgggcagcgttttcctggagaaccgtgaatttgttatggtgctgaccaccagcatcgcggtgctgattggctgcgtggttgtgttcatctggcgtcgtagcaccggtaacaaggcgaaaagcattgagccgccgaaacgtgttgtggaaaagctgagcgatgaggcggaagtggacgatggcacccgtaaagttaccatcttctttggcacccaaaccggcaccgcggagggttttgcgaaagcgattgcggaggaagcgaaagtgcgttacgaaaaggcgaaatttaagattgttgacatggacgattacgcgcaagacgatgacgagtatgaggaaaagctgaagaaagaaaccctggcgctgttctttctggcgacctacggtgatggtgaaccgaccgacaacgcggcgcgtttctataaatggtttctggagggtgacgaaaaggaagagggttggctgcgtaacctggaatacgcggttttcggtctgggcaaccgtcagtatgagcactttaacaaagttgcgatcgaagtggatgacaagctggcggatttcggtggcaaacgtctggttaaagtgggtctgggcgatgacgatcaatgcattgaggacgattttaccgcgtggaaagaggaactgtggccggcgctggacgaactgctgcgtggcgacgatgacaccaccgtgagcaccccgtacaccgcggcggttctggagtatcgtgttgtgatccacgatccgctggacgcgagcgtggatgaaaagaaatggcacaacgttaacggtcacgcgattgtggacgcgcagcacccggttcgtagcaacgttgcggtgcgtaaagagctgcacaccccggtgagcgatcgtagctgcacccacctggagttcgacatcagcggcaccggtgtggcgtacgaaaccggtgatcacgttggcgtgtattgcgagaacctgagcgaaaccgttgaggaagcggtgcgtctgctgggtctgagcccggatacctacttcagcgttcacaccgatgacgaggacggtaaaccgctgagcggcagcagcctgccgccgacctttccgccgtgcaccctgcgtaccgcgattgcgcgttatgcggacgtgctgagcagcccgaagaaaagcgttctgctggcgctggcggcgcatgcgagcaacccgagcgaagcggatcgtctgcgtcacctggcgagcccggcgggtaaagacgagtacagcgaatgggtgattgcgagccagcgtagcctgctggaagtgatggcggagttcccgagcgcgaagccgccgatcggtgtgttctttgcggcgattgcgccgcgtctgcaaccgcgtttttatagcatcagcagcagcccgcgtatggcgccgagccgtattcacgttacctgcgcgctggtgaacgacaaaatgccgaccggtcgtatccaccgtggcgtgtgcagcacctggatgaaaaacagcgttccgctggagaagagccaggattgcagctgggcgccgatcttcgtgcgtcaaagcaacttcaagctgccggcggacaacaaggttccgatcattatgattggtccgggcaccggtctggcgccgttccgtggtttcctgcaagaacgtctggcgctgaaagaggacggtgcggaactgggtccgagcgtgctgttctttggttgccgtaaccgtcaaatggattacatctatgaggacgaactgaaccacttcgtgaacagcggcgcgctgagcgagctgattgttgcgtttagccgtgagggtccgaccaaagaatacgttcagcacaaaatgatggaaaaggcgagcgacatctggaacatgattagccagggtgcgtacatctatgtgtgcggtgatgcgaagggcatggcgcgtgacgttcaccgtaccctgcacaccattctgcaagagcaaggtagcctggatagcagcaaagcggaaggcatggtgaagaacctgcaactgaacggtcgttatctgcgtgacgtttggtaa
NOMT	Atggtgagcccggtggtacaccgtcacgccgccggcggcggcagcggcggcgacgacgacgaccaggcttgcatgtacgcgctggagctcctgggagggtcggtcgtctccatgacactcaaggcggcgatcgagctcggcctcgtcgacgagctcctcgccgccgccggcgccgccgtgaccgcggaggagctagctgcgcggctccggctccctgctgcggtcgccgccgcggcggcggtggaccgcatgctgcggctcctcgcctcgtacggcgtcgtcaggtgcgcgacggaggccgggcccgacggcaaggcccgccggagctacgccgcggcgccggtgtgcaaatggctcgccgccgggagcagcagcggcgaagggtccatggctccgcttgggctgctcaacctcgacaaggtgttcatggagaactggtactacctgaaggaggcagtgtcggagggagggacagcattcgacaaggcgtacggcacgtccttgttccagt

	acctgggccaggacggcaacgagccctccaacacgctcttcaaccaggccatggccagccactccgtcgtcatcaccaacaagctgctccaattcttccgcggcttcgacgccggcgccggcgtggatgtg<br>ctcgtcgacgtcggcggcggcgtcggcgccacgctgcggatgatcaccgctcgccacccccacctgcgtggcgtcaactacgaccttccccatgtcatcgcgcaggcgccgccagttgaaggtgtggagc<br>atatcggaggcagcatgtttgaccatgttcccagtggaagcgcaatcttgctcaagtggattctgcacttgtggggggacgaggagtgcgtgaagatcctgaagaactgctacaaggcgctgccggcgaagg<br>ggaaggtgatcttggtggagtatgtgctcccggcgagcccggaggcgacgctggcggcgcaggaagcgttccggctcgacgtcatgatgctgaaccgcctcgccggcggcaaggagaggacgcagca<br>ggagttcaccgacctcgccgtcgacgccggcttctccggcgactgcaagcctacctacatcttcaccaacgtctgggctctcgagttcacaaagtaa
PfOMT3	atgaagaattcatcaacggatgaagatttatccatattcgcgatgcaagtggctacttcttccatagtccccagagctctgaaagctgtcatagaactagacctgctggagatgatgaagaaggccggccgtccc<br>ctttccacgtcagaaatggccgcccagattcaggccaccaacccggaggccgccctcatgattgacaggatccttcgagttctcatcgccagcaacattctagaatgcaccactgctgcctctcacggtggcg<br>ctgagcggctttattctttggctccggtttgcaagttcttcaccaagaatgatgatggtgtttcttgggctccattgtttctcatgatccaagacagagtcttcacagaagcctgggatcatgtaaaggatgcaatagtt<br>gaaggaggaatcccgttcaatatagcccatggaatgagtgggttcgaatacccggcaaccgacccgagatataacaagattttcaaccaagccatgtctgatgaatccaccatgtttatgcataaaattcttgaat<br>tatatgatggatttgacggtttgaaatctgttgtggatgttggtggtggaattggagcttcactaaagatgattataaccaagtatccatctattcaggccatcaatttcgatttgccccatgtcatccaaaatgctccat<br>ctcatcctgggttggagcacagaagtggagacatgtttgttagtgtgcctacaggagatgccattttgttgaagtggattatccataattggagcgatgggcattgcttaaaactcctgaaaaactgctacgaagca<br>cttccagaaaagggaaaagtgataatcgcagatcggattcttccggaaacagagaattataaggaggcatcggcgtcggtagacttggccggcgacgcgctcatgttaacgttgttcaccggtgggaaggag<br>agggcagaagcagaatttcaagctttggcaaaagcatctggtttcaaacatttccgtaaagtttgttgtgctttcagcacttggatcatggaactctacaaataa

Table S9. Promoter sequences used in this study.

Promoters	Sequences	References
$P_{\mathrm{PalsRBACE-UTRrpsT}}$	agcaacatctatcatctaaaaaaccagaaaaacaaataacatcatgtttttaaactaattaaatgaccatcactacgtaacgagtgcc<br>ggcacattaacggcgcttatttgcacaaatccattgacaaaagaaggctaaaagggcatattcctcggcctttgaattgtccatatag<br>aacacatttgggagttggacc	[5a]
$P_{\mathrm{grpE-UTRrpsT}}$	gattgatgacaatgtgagtgcttcccttgaaaccctgaaactgatccccataataagcgaaccatcactacgtaacgagtgccggc<br>acattaacggcgcttatttgcacaaatccattgacaaaagaaggctaaaagggcatattcctcggcctttgaattgtccatatagaac<br>acatttgggagttggacc	[5a]
$P_{\mathrm{dnaKJ-UTRrpsT}}$	gcacaaaaaatttttgcatctccccccttgatgacgtggtttacgaccccatttagtagtcaaccgcagtgagtgagtctgcaaaaaaa<br>tgaaattgggcagttgaaaccagacgtttcgcccctattacagactcacaaccacatgatgaccgaataccatcactacgtaacga<br>gtgccggcacattaacggcgcttatttgcacaaatccattgacaaaagaaggctaaaagggcatattcctcggcctttgaattgtcc<br>atatagaacacatttgggagttggacc	[5a]
$P_{\mathrm{ssrA-UTRrpsT}}$	attggctatcacatccgacacaaatgttgccatcccattgcttaatcgaataaaaatcaggctacatgggtgctaaatctttaacgata<br>acgccattgaggctggtcatggcgctcataaatctggtatacttacctttaccatcactacgtaacgagtgccggcacattaacggc<br>gcttatttgcacaaatccattgacaaaagaaggctaaaagggcatattcctcggcctttgaattgtccatatagaacacatttgggagt<br>tggacc	[5a]
$P_{\mathrm{UTRssrA-PUTRinfC-rplT}}$	attggctatcacatccgacacaaatgttgccatcccattgcttaatcgaataaaaatcaggctacatgggtgctaaatctttaacgata<br>acgccattgaggctggtcatggcgctcataaatctggtatacttacctttacacattgcgggcattcgtgttaaagcagacttgagaa<br>atgagaagattggctttaaaatccgcgagcacactttgcgtcgcgtcccatatatgctggtctgtggtgataaagaggtggaatcag<br>gcaaagttgccgttcgcacccgccgtggtaaagacctgggaagcatggacgtaaatgaagtgatcgagaagctgcaacaagag<br>attcgcagccgcagtcttaaacaattggaggaataaggt	[5a]
P_{rrnA}	cagaaaattattttaaatttcctcttgtcaggccggaataactccctataatgcgccaccactgacacggaacaacggcaaacacgc<br>cgccgggtcagcggggttctcctgagaactccggcagagaaagcaaaaataaatgcttgactctgtagcgggaaggcgtattat<br>gcacaccccgcgccgctgagaaaaagcgaagcggcactgctctttaacaatttatcagacaatctgtgtgggcactcgaagatac<br>ggattcttaacgtcgcaagacgaaaaatgaataccaagtctcaagagtgaacacgtaattcattacgaagtttaattctttgagcgtc<br>aaactttt	[5a]
P_{dnaKJ}	gcacaaaaaatttttgcatctccccccttgatgacgtggtttacgaccccatttagtagtcaaccgcagtgagtgagtctgcaaaaaaa<br>tgaaattgggcagttgaaaccagacgtttcgcccctattacagactcacaaccacatgatgaccgaatatatagtggagacgtttag	[5a]
P_{sdhCDAB}	cgacaaactatatgtaggttaattgtaatgattttgtgaacagcctatactgccgccaggtctccggaacaccctgcaatcccgagc<br>cacccagcgttgtaacgtgtcgttttcgcatctggaagcagtgttttgcatgacgcgcagttatagaaaggacgctgtctgacccgc<br>aagcagaccggaggaaggaaatcccgacgtctccaggtaacagaaagttaacctctgtgcccgtagtccccagggaataataag<br>aacagcatgtgggcgttattc	[5a]
P_{yibN}	aaattgcattccagttaacgcgctggctgtatttgccgcaccgcgcaggtatactcctttcctggtttttttaatcactacgtcgggagtt<br>gttaccccc	[5a]
P_{ssrA}	attggctatcacatccgacacaaatgttgccatcccattgcttaatcgaataaaaatcaggctacatgggtgctaaatctttaacgata<br>acgccattgaggctggtcatggcgctcataaatctggtatacttacctttacacatt	[5a]
P_{rpmH}	cgccgcatattggaaaatttaatgaccatagacaaaaattggcttaatcgatctaataaagatccaggacgatccttgcgctttaccc<br>atcagcccgtataatcctccacccggcgcgccatgctggtttccactggtgtgaggtcgtacattttccctgcgaaaaggtgcgga<br>aaagcgcggtaaataaggaaagagaattgactccggagtgtacaattattacaatccggcctctttaatcacccatggcttcggtgt<br>ccatcgtttcatttttcggcggatatccaataaagccattgaatttattcaagtttaggtagaaatcgcc	[5a]
P_{RacR}	ctggacatctctcggcaggcaattgacgtacccctttgatccattgatttacgcttggaggtgatacacctaaaagcctagccattgc<br>tgattgcccaccgacaacagcacaagcttgcttgaatgaatagttctctttttttcatcgaatgaactccaaaaacacacagaaatatta<br>ggcgacgcctaacgcaattgtcaataggctgtgcctaatgcagtaagggtagggattgcctaatgtaatgcgcataggagaatatt<br>aagca	[5a]
P_{yccFS}	cagagaaaacgtgctccagcccaaaaaaacgttacaattgccgcccattactgaaaaccacagtaaagcgaggtttt	[5a]
P_{alsRBACE}	agcaacatctatcatctaaaaaaccagaaaaacaaataacatcatgtttttaaactaattaaatgaaataaaattttaagccactcgcc<br>attgttcacaataaaataaactttataaattttattttttgtgaagtcgccagcatcttttctgttcttgctgtggtgatatagtggcgtcttc<br>aattcaaggacaagagaacgtg	[5a]

P_{trxA}	gcgaagtcggaaaacttctgttctgttaaatgtgttttgctcatagtgtggtagaatatcagcttactattgctttacgaaagcgtatccg<br>gtgaaataaagtcaacctttagttggttaatgttacaccaacaacgaaaccaacacgccaggcttattcctgtggagttatat	[5a]
P_{grpE}	gattgatgacaatgtgagtgcttcccttgaaaccctgaaactgatccccataataagcgaagttagcgagatgaatgcgaaaaaaa<br>cgcggagaaattc	[5a]
P_{talB}	cctggcgataaccgtcttgtcggcggttgcgctgacgttgcgtcgtgatatcatcagggcagaccggttacatccccctaacaagc<br>tgtttaaagagaaatactatc	[5a]
P_{mdh}	aaattaattgttatcaaattgatgttgttttggctgaacggtagggtatattgtcaccacctgttggaatgttgcgctaatgcataagcga<br>ctgttaattacgtaagttaggttcctgattacggcaattaaatgcataaacgctaaacttgcgtgactacacattcttgagatgtggtca<br>ttgtaaacggcaattttgtggattaaggtcgcggcagcggagcaacatatcttagtttatcaatataataaggagtttagg	[5a]
P_{rplNXE}	ctcagaaatgagccgtttatttttctacccatatccttgaagcggtgttataatgccgcgccctcgatatggggatttttaacgacctg<br>attttcgggtctcagtagtagttgacattagcggagcactaaa	[5a]
P_{rspU}	atgcgggttgatgtaaaactttgttcgcccctggagaaagcctcgtgtatactcctcacccttataaaagtccctttcaaaaaaggcc<br>gcggtgctttacaaagcagcagcaattgcagtaaaattccgcaccattttgaaataagctggcgttgatgccagcggcaaaccgaa<br>ttaatcaaaggtgagaggcac	[5a]
$P_{infC-rplT}$	gcgggcattcgtgttaaagcagacttgagaaatgagaagattggctttaaaatccgcgagcacactttgcgtcgcgtcccatatatg<br>ctggtctgtggtgataaagaggtggaatcaggcaaagttgccgttcgcacccgccgtggtaaagacctgggaagcatggacgta<br>aatgaagtgatcgagaagctgcaacaagagattcgcagccgcagtcttaaacaattggaggaataaggt	[5a]
P_{1-33}	tggtaaaaaaaactgtgggaaaatttgttaaaacttggtaaaatagccta	[5b]
P_{1-17}	atgcactgttaccctttgtaatattgttcagtatggcctatgctacgcat	[5b]
P_{1-2}	ggtcaacaattatagttgattatcgcgtaaaagtatgctacccttaagtt	[5b]
P_{2-19}	gtcgtcgcaatgacgttgaatatctggtgattaattgctaaactaacgtt	[5b]
P_{2-22}	gaactggttaaggtgatggacaacgggtggtaatatgctatactaaaggc	[5b]
P_{2-21}	gccgtcagtttagccagaccgccgaaaatttccaacacttctgaaacacc	[5b]
P_{1-26}	acgcacactttcacgtcgcaaaatggtacactattggctatgctacgcac	[5b]
P_{1-9}	tgtttaaattttgatttaagtcttatcggaggctatgctatttgcgattt	[5b]
P_{1-45}	gagaacttctatgggtggctaatgtgacgaaagaaggtatgctggtcacc	[5b]
P_{1-16}	ttatgataagttcactgttggctaaagggagtgtaagtcataatgaactt	[5b]
P_{1-57}	cgaatcactagatctctggcattgatttaaataagataaaagtatgactt	[5b]
P_{23119}	ttgacagctagctcagtcctaggtataatgctagc	Anderson promoter
P_{23100}	ttgacggctagctcagtcctaggtacagtgctagc	Anderson promoter
P_{23104}	ttgacagctagctcagtcctaggtattgtgctagc	Anderson promoter
P_{23107}	tttacggctagctcagccctaggtattatgctagc	Anderson promoter
P_{23110}	tttacggctagctcagtcctaggtacaatgctagc	Anderson promoter
P_{23105}	tttacggctagctcagtcctaggtactatgctagc	Anderson promoter
P_{23113}	ctgatggctagctcagtcctagggattatgctagc	Anderson promoter
P_{23103}	ctgatagctagctcagtcctagggattatgctagc	Anderson promoter
P_{23112}	ctgatagctagctcagtcctagggattatgctagc	Anderson promoter

Note: promoters from *Zhou et al* [5a] were obtained by amplifying the genome of BL21(DE3). Other promoters were designed in primers and obtained by PCR reaction. Sequences of Anderson promoters were obtained from website (https://parts.igem.org/Promoters/Catalog/Anderson).

Table S10. Flavonoid bioactivates in this study.

Flavonoids	Bioactivates	References
Resveratrol	antioxidant, anti-inflammation, and anticancer	[26]
Genistein	ati-aging, anticancer and prevent neurodegenerative diseases	[12, 27]

Sakuranetin	antidiabetic, antiviral, antimicrobial and anti-inflammatory	[13b, 28]
Hesperetin	antioxidant, anti-inflammatory, and neuroprotection	[14, 29]

Table S11. The corresponding information for flavonoid biosynthesis in this study.

Compounds	Strategy	Culture styles	Titer (mg/L)	References
resveratrol	One strain without malonyl-CoA supply module	Culture strain in MOPS media at 30°C; add 1 mM IPTG when OD_{600} reaches 0.60-0.80; incubate 30 °C for 2 days.	82.06	this study
resveratrol	Co-culture (one strain for **naringenin production**, one strain for **malonyl-CoA supply**) a*	Incubate strain at 37°C; add 1 mM IPTG and fresh MOPS media when OD_{600} reaches 1.65; culture the media at 30°C for two days.	35.02	[30]
resveratrol	One strain containing **tyrosine supply module** b*	Culture strain in LB media; collect strain when OD600 reaches 1.0; resuspend in M9 medium with 1 g/L glycerol 1 mM IPTG; incubated at 30 °C for 48 h.	80.40	[31]
genistein	One strain without tyrosine supply module	Culture strain in MOPS media at 30°C; add 1 mM IPTG when OD_{600} reaches 0.60-0.80; incubate 30 °C for 2 days.	77.85	this study
genistein	Co-culture (one strain for **p-coumaric acid;** one strain for **naringenin biosynthesis**; one strain for **genistein biosynthesis**) c*	Separately incubate in LB media for 12h; transfer strains to M9Y media e* in corresponding inoculation ratios based on the individual OD; add 0.1 mM IPTG; Culture at 30°C.	60.80	[12]
sakuranetin	Overexpress NOMT in our naringenin chassis	Culture strain in MOPS media at 30°C; add 1 mM IPTG when OD_{600} reaches 0.60-0.80; incubate 30 °C for 2 days.	223.39	this study
sakuranetin	Overexpress NOMT in the BL21(DE3) with higher tyrosine supply module d*	Culture strain in LB medium at 37°C; add 1mM IPTG when OD600 reaches 0.8; incubate 18 h at 18 °C; suspend strain and transfer them into M9 medium; regulate the OD600 to 3.0, add 2 % glucose, 1 mM IPTG, and 300 µM p-coumaric acid; culture 30°C for 24 h.	40.10	[13a]
sakuranetin	Overexpress PfOMT3 in our naringenin chassis	Culture strain in MOPS media at 30°C; add 1 mM IPTG; incubate 30 °C for two days.	57.19	this study
sakuranetin	Overexpress PfOMT3 in BL21(DE3)	Culture strains in LB medium at 37°C; add 2% (v/v) of LB strain culture into TB medium; add 0.1 mM IPTG when OD_{600} reaches 0.8-1 f*; incubate 25°C; add 350 mg/L (2S)-naringenin after 3 h and 13 h incubation.	173.61	[13b]
hesperetin	One strain	Culture strain in MOPS media at 30°C; add 1 mM IPTG when OD_{600} reaches 0.60-0.80; incubate 30 °C for 2 days.	82.50	this study
hesperetin	Co-culture (one strain convert naringenin to **eriodictyol**, which is catalyzed to **hesperetin** by another strain)	Culture two strains in LB medium at 37°C; add 0.2 mM IPTG until reaches 0.6–0.8; harvest the strains after 10 h inducible time; resuspend the cell in 50 mM Tris-HCl buffer with naringenin and 5-aminolevulinic acid.	37.10	[14]

a*: malonate synthetase (matB) and malonate carrier protein (matC) was overexpressed for malonyl-CoA supply.
b*: higher tyrosine supply module is BL21(DE3)ΔtyrRΔpheAΔicdAΔfumC::aroGfbr::ppsA::tktA::tyrAfbr. Higher tyrosine supply can be obtained by targeting tyrR, pheA, icdA and fumC genes and overexpressing aroGfb, ppsA, tktA and tyrAfbr.
c*: p-coumaric acid module is BL21(DE3) ΔptsGΔtyrRΔpheA:: aroGfbr::tyrAfbr::aroE and ppsA::tktA::glk. Higher tyrosine supply can be obtained by targeting ptsG, tyrR, and pheA genes and overexpressing aroGfb, tyrAfbr, aroE, ppsA, tktA and glk.
d*: higher tyrosine supply module is BL21(DE3) ΔtyrRΔpheAΔicdA::aroGfbr::ppsA::tktA::tyrAfbr. Higher tyrosine supply can be obtained by targeting tyrR, pheA and icdA genes and overexpressing aroGfb, ppsA, tktA tyrAfbr.
e*: M9Y medium (1g/L yeast extract, 17.1 g/L $Na_2HPO_4 \cdot 12H_2O$, 3.0 g/L KH_2PO_4, 0.5 g/L NaCl, 1.0 g/L NH_4Cl, 5.0 mM $MgSO_4$, and 0.1 mM $CaCl_2$)
f*: LB medium (10 g/L tryptone, 5 g/L yeast extract, 10 g/L NaCl); TB medium (11.8 g/L SELECT peptone, 23.6 g/L yeast extract, 9.4 g/L K_2HPO_4, 2.2 g/L KH_2PO_4, 4 mL/L glycerol)

References

[1] a) S. Zhou, Y. Lyu, H. Li, M. A. G. Koffas, J. Zhou, *Biotechnol Bioeng* **2019**, *116* (6), 1392, https://doi.org/10.1002/bit.26941; b) S. Zhou, S. F. Yuan, P. H. Nair, H. S. Alper, Y. Deng, J. Zhou, *Metab Eng* **2021**, *67*, 41, https://doi.org/10.1016/j.ymben.2021.05.007.
[2] S. Zhou, P. Liu, J. Chen, G. Du, H. Li, J. Zhou, *Appl Microbiol Biotechnol* **2016**, *100* (24), 10443, https://doi.org/10.1007/s00253-016-7672-8.
[3] M. Alberstein, M. Eisenstein, H. Abeliovich, *The Plant journal : for cell and molecular biology* **2012**, *69* (1), 57, https://doi.org/10.1111/j.1365-313X.2011.04770.x.
[4] D. Kong, S. Li, C. D. Smolke, *Science advances* **2020**, *6* (44), eabd1143, https://doi.org/10.1126/sciadv.abd1143.
[5] a) S. Zhou, R. Ding, J. Chen, G. Du, H. Li, J. Zhou, *Acs Synth Biol* **2017**, *6* (6), 1065, https://doi.org/10.1021/acssynbio.7b00006; b) Y. Wang, H. Wang, L. Wei, S. Li, L. Liu, X. Wang, *Nucleic Acids Res* **2020**, *48* (12), 6403, https://doi.org/10.1093/nar/gkaa325.
[6] T. J. Lambert, *Nat Methods* **2019**, *16* (4), 277, https://doi.org/10.1038/s41592-019-0352-8.
[7] M. HamediRad, S. Weisberg, R. Chao, J. Lian, H. Zhao, *Acs Synth Biol* **2019**, *8* (5), 1047, https://doi.org/10.1021/acssynbio.8b00480.
[8] P. Bernard, M. Couturier, *Journal of Molecular Biology* **1992**, *226* (3), 735, https://doi.org/https://doi.org/10.1016/0022-2836(92)90629-X.
[9] M. Van Brempt, A. I. Peeters, D. Duchi, L. De Wannemaeker, J. Maertens, B. De Paepe, M. De Mey, *Microb Cell Fact* **2022**, *21* (1), 49, https://doi.org/10.1186/s12934-022-01775-8.
[10] J. Li, C. Tian, Y. Xia, I. Mutanda, K. Wang, Y. Wang, *Metab Eng* **2019**, *52*, 124, https://doi.org/10.1016/j.ymben.2018.11.008.
[11] Y. Zhou, G. Li, J. Dong, X.-h. Xing, J. Dai, C. Zhang, *Metabolic Engineering* **2018**, *47*, 294, https://doi.org/https://doi.org/10.1016/j.ymben.2018.03.020.
[12] X. Liu, L. Li, G.-R. Zhao, *Acs Synth Biol* **2022**, *11* (5), 1746, https://doi.org/10.1021/acssynbio.1c00590.
[13] a) M.-J. Kim, B.-G. Kim, J.-H. Ahn, *Appl Microbiol Biot* **2013**, *97* (16), 7195, https://doi.org/10.1007/s00253-013-5020-9; b) Q. Sun, S. Gao, S. Yu, P. Zheng, J. Zhou, *Synth Syst Biotechnol* **2022**, *7* (4), 1117, https://doi.org/10.1016/j.synbio.2022.07.004.
[14] J. Liu, M. Tian, Z. Wang, F. Xiao, X. Huang, Y. Shan, *Journal of Biotechnology* **2022**, *347*, 67, https://doi.org/https://doi.org/10.1016/j.jbiotec.2022.02.008.
[15] S. Y. Jun, S. A. Sattler, G. S. Cortez, W. Vermerris, S. E. Sattler, C. Kang, *Plant Physiol* **2018**, *176* (2), 1452, https://doi.org/10.1104/pp.17.01608.
[16] J. Rosler, F. Krekel, N. Amrhein, J. Schmid, *Plant Physiology* **1997**, *113* (1), 175, https://doi.org/10.1104/pp.113.1.175.
[17] K. T. Watts, B. N. Mijts, P. C. Lee, A. J. Manning, C. Schmidt-Dannert, *Chem Biol* **2006**, *13* (12), 1317, https://doi.org/10.1016/j.chembiol.2006.10.008.
[18] J. A. Kyndt, T. E. Meyer, M. A. Cusanovich, J. J. Van Beeumen, *FEBS Letters* **2002**, *512* (1-3), 240, https://doi.org/https://doi.org/10.1016/S0014-5793(02)02272-X.
[19] Z. Jin, J. Wungsintaweekul, S.-H. Kim, J.-H. Kim, Y. Shin, D.-K. Ro, S.-U. Kim, *Biochemical Journal* **2020**, *477* (1), 61, https://doi.org/10.1042/bcj20190527.
[20] Z. Li, S. K. Nair, *Structure* **2015**, *23* (11), 2032, https://doi.org/10.1016/j.str.2015.08.012.
[21] X. Y. Liu, P. P. Wang, Y. F. Wu, A. X. Cheng, H. X. Lou, *Molecules* **2018**, *23* (3), https://doi.org/10.3390/molecules23030595.
[22] Z. B. Li, C. F. Li, J. Li, Y. S. Zhang, *Biol Pharm Bull* **2014**, *37* (1), 113, https://doi.org/10.1248/bpb.b13-00633.

[23] T. Waki, R. Mameda, T. Nakano, S. Yamada, M. Terashita, K. Ito, N. Tenma, Y. Li, N. Fujino, K. Uno, S. Yamashita, Y. Aoki, K. Denessiouk, Y. Kawai, S. Sugawara, K. Saito, K. Yonekura-Sakakibara, Y. Morita, A. Hoshino, S. Takahashi, T. Nakayama, *Nature Communications* **2020**, *11* (1), 870, https://doi.org/10.1038/s41467-020-14558-9.
[24] J. M. Jez, M. E. Bowman, J. P. Noel, *Proc Natl Acad Sci U S A* **2002**, *99* (8), 5319, https://doi.org/10.1073/pnas.082590499.
[25] Y. Shen, X. Li, T. Chai, H. Wang, **2016**, *6* (6), 610, https://doi.org/https://doi.org/10.1002/2211-5463.12072.
[26] a) J. A. Baur, D. A. Sinclair, *Nature Reviews Drug Discovery* **2006**, *5* (6), 493, https://doi.org/10.1038/nrd2060; b) B. Ren, M. X.-Y. Kwah, C. Liu, Z. Ma, M. K. Shanmugam, L. Ding, X. Xiang, P. C.-L. Ho, L. Wang, P. S. Ong, B. C. Goh, *Cancer letters* **2021**, *515*, 63, https://doi.org/https://doi.org/10.1016/j.canlet.2021.05.001.
[27] V. Mukund, D. Mukund, V. Sharma, M. Mannarapu, A. Alam, *Critical Reviews in Oncology/Hematology* **2017**, *119*, 13, https://doi.org/https://doi.org/10.1016/j.critrevonc.2017.09.004.
[28] M. Stompor, *Nutrients* **2020**, *12* (2), 513.
[29] a) H. Parhiz, A. Roohbakhsh, F. Soltani, R. Rezaee, M. Iranshahi, *Phytother Res* **2015**, *29* (3), 323, https://doi.org/https://doi.org/10.1002/ptr.5256; b) M. Ikram, T. Muhammad, S. U. Rehman, A. Khan, M. G. Jo, T. Ali, M. O. Kim, *Molecular Neurobiology* **2019**, *56* (9), 6293, https://doi.org/10.1007/s12035-019-1512-7.
[30] J. Wu, P. Liu, Y. Fan, H. Bao, G. Du, J. Zhou, J. Chen, *J Biotechnol* **2013**, *167* (4), 404, https://doi.org/10.1016/j.jbiotec.2013.07.030.
[31] J. Y. Park, J.-H. Lim, J.-H. Ahn, B.-G. Kim, *Applied Biological Chemistry* **2021**, *64* (1), 20, https://doi.org/10.1186/s13765-021-00595-5.
